# Pathogenic function of bystander-activated memory-like CD4^+^ T cells in autoimmune encephalomyelitis

**DOI:** 10.1038/s41467-019-08482-w

**Published:** 2019-02-12

**Authors:** Hong-Gyun Lee, Jae-Ung Lee, Do-Hyun Kim, Sangho Lim, Insoo Kang, Je-Min Choi

**Affiliations:** 1 Department of Life Science, College of Natural Sciences, Hanyang University, Seoul, 04763 Republic of Korea; 2 Research Institute for Natural Sciences, Hanyang University, Seoul, 04763 Republic of Korea; 3 Department of Internal Medicine, Yale University School of Medicine, New Haven, CT 06510 USA

## Abstract

T cells generate antigen-specific immune responses to their cognate antigen as a hallmark of adaptive immunity. Despite the importance of antigen-specific T cells, here we show that antigen non-related, bystander memory-like CD4^+^ T cells also significantly contribute to autoimmune pathogenesis. Transcriptome analysis demonstrates that interleukin (IL)-1β- and IL-23-prime T cells that express pathogenic T_Η_17 signature genes such as RORγt, CCR6, and granulocyte macrophage colony-stimulating factor (GM-CSF). Importantly, when co-transferred with myelin-specific 2D2 TCR-transgenic naive T cells, unrelated OT-II TCR-transgenic memory-like T_H_17 cells infiltrate the spinal cord and produce IL-17A, interferon (IFN)-γ, and GM-CSF, increasing the susceptibility of the recipients to experimental autoimmune encephalomyelitis in an IL-1 receptor-dependent manner. In humans, IL-1R1^high^ memory CD4^+^ T cells are major producers of IL-17A and IFN-γ in response to IL-1β and IL-23. Collectively, our findings reveal the innate-like pathogenic function of antigen non-related memory CD4^+^ T cells, which contributes to the development of autoimmune diseases.

## Abstract (web summary)

T cells express specific T cell receptors (TCR) to recognise antigens and initiate adaptive immune responses. Here the authors show, in a mouse model of autoimmune encephalomyelitis, that memory-like CD4 T cells expressing unrelated TCR can also infiltrate the spinal cord and contribute to autoimmunity.

## Introduction

Multiple sclerosis (MS) is an unpredictable, chronic, demyelinating, human autoimmune disease caused by the induction of inflammation in the central nervous system (CNS)^1^. Studies of experimental autoimmune encephalomyelitis (EAE), a model of multiple sclerosis (MS), have demonstrated that CNS-invading myelin-specific T_H_1 and T_H_17 cells are the major mediators of autoimmune neuroinflammation^2–4^. T_H_17 cells are categorized into two functionally distinct subsets: non-pathogenic T_H_17 and pathogenic T_H_17 cells^5^. T_H_17 cells differentiate in the presence of transforming growth factor (TGF)-β and interleukin (IL)-6 produce IL-17A and IL-10, which are not pathogenic^6^. However, further stimulation with IL-1β and IL-23 induces highly encephalitogenic T_H_17 cells, which have been shown to express signature genes, including RORγt, T-bet, IL-17A, IL-22, and granulocyte macrophage colony-stimulating factor (GM-CSF)^5,7–9^.

Recently, IL-17-producing innate-like lymphocytes, such as gamma delta (γδ) T cells, invariant natural killer T cells, and innate lymphoid cells were shown to be important for responding to the pro-inflammatory cytokines IL-1β and IL-23, by producing IL-17 in an antigen-nonspecific manner^10–13^. The ability of innate-like lymphocytes to produce innate IL-17 has been shown to be critical in many autoimmune disease models, including experimental autoimmune encephalomyelitis (EAE)^14,15^ and inflammatory bowel disease^16,17^. CD4^+^ T lymphocytes respond to their specific cognate antigen and further differentiate into distinct subsets of helper T cells, including T_H_1, T_H_2, and T_H_17, as defined by their pattern of effector cytokine production^18^. However, differentiated CD4^+^ T cells can respond directly to pro-inflammatory cytokines by producing innate effector cytokines. IL-1 family cytokines (IL-18, IL-33, IL-1β), along with the STAT activator cytokines (IL-12, IL-2, IL-23), were shown to promote effector cytokine production by T_H_1, T_H_2, and T_H_17 cells^19^. Moreover, IL-33-dependent IL-13 production by memory T_H_2 cells has been shown to contribute to allergic inflammation and protect against early helminth infection^20^. These findings demonstrate that the innate-like capacity of CD4^+^ T lymphocytes, which is correlated with innate-like lymphocytes, produce effector cytokines in response to pro-inflammatory cytokines. However, whether the innate immunological function of CD4^+^ T lymphocytes contributes to the pathogenicity of autoimmune diseases remains unclear.

CD4^+^ T lymphocytes specific for nonmyelin proteins have been proposed to invade the CNS^21,22^, regardless of their specificity for CNS antigens, thus providing encephalitogenic potential^23,24^. Furthermore, in an EAE model, most CNS-infiltrating CD4^+^ T cells were found to be myelin oligodendrocyte glycoprotein (MOG)-nonspecific^25–27^. Although nonmyelin-specific T cells have been associated with the pathogenesis of autoimmune disorders, the precise mechanism is unknown.

Here, we hypothesized that antigen non-related CD4^+^ T cells contribute to autoimmune disease pathogenesis in response to pro-inflammatory cytokines. We first screened for pro-inflammatory cytokines capable of initiating innate effector cytokine production by CD4^+^ T cells. We found that memory-like CD4^+^ T cells, but not naive CD4^+^ T cells, produced IL-17A and interferon (IFN)-γ in response to IL-1β and IL-23 in the absence of T-cell receptor (TCR) engagement. Bystander activation of memory-like CD4^+^ T cells increased the expression of pathogenic T_H_17 signature genes, including RORγt, CCR6, and GM-CSF. Furthermore, TCR-transgenic (OT-II) memory-like T_H_17 cells were shown to contribute to EAE pathogenicity regardless of antigen specificity by infiltrating and producing IL-17A, IFN-γ, and GM-CSF in the spinal cord in an IL-1R1-dependent manner. Taken together, our findings demonstrate the importance of the TCR-independent innate-like pathogenic role of bystander-activated memory CD4^+^ T cells in autoimmune encephalomyelitis.

## Results

### TCR-independent CD4 T cells activation via IL-1β and IL-23

**Fig. 1:**
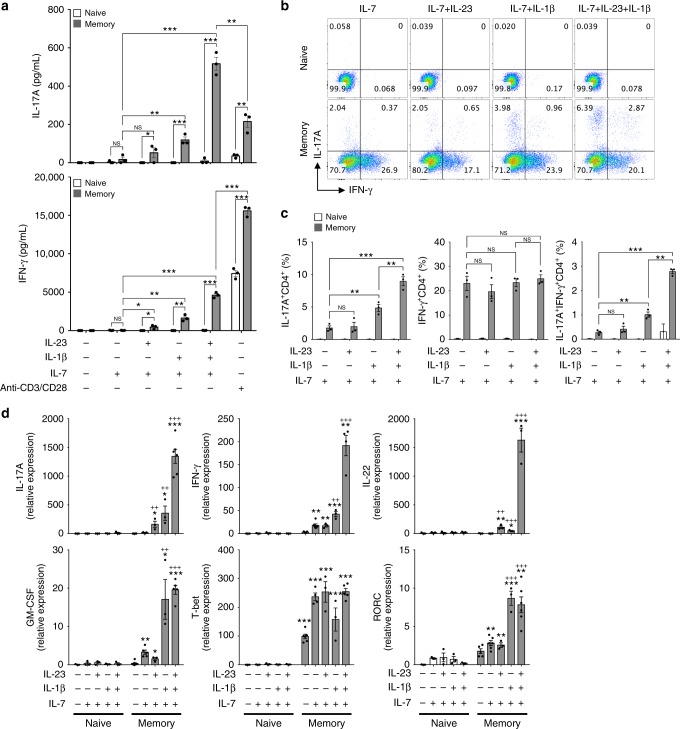
Memory-like CD4^+^ T cells respond to IL-1β and IL-23 by producing IL-17A and IFN-γ in the absence of TCR engagement. Naive (CD4^+^CD25^−^CD62L^high^CD44^low^) and memory-like (CD4^+^CD25^-^CD62L^low^CD44^high^) T cells were stimulated with IL-1β and/or IL-23 in the presence of IL-7 or anti-CD3/CD28 for 5 days. **a** IL-17A and IFN-γ concentrations in the supernatants were measured by ELISA. **b**, **c** The frequencies of IL-17A and IFN-γ-producing cells were analyzed by flow cytometry. **d** mRNA levels of transcription factors and cytokines were analyzed by quantitative real-time PCR. *p < 0.05, **p < 0.01, ***p < 0.001 versus naive CD4^+^ T cells plus IL-7; ^+^*p* < 0.05, ^++^*p* < 0.01, ^+++^*p* < 0.001 versus memory-like CD4^+^ T cells plus IL-7. Data are presented as the mean ± SEM of at least three independent experiments

To examine the innate-like capacity of CD4^+^ T lymphocytes, CD4^+^CD25^−^ T cells were sorted by fluorescence-activated cell sorting (FACS) and cultured in the presence of pro-inflammatory cytokines, including tumor necrosis factor (TNF), IL-6, IL-23, IL-12, and IL-1β in the absence of TCR stimulation. Additionally, IL-7 was added to the culture medium for T-cell survival and maintenance^28,29^. Consistent with previous results^30,31^, we found that IL-12 promoted IFN-γ production, which further synergized with TNF and IL-1β (Supplementary Fig. 1a). Interestingly, the pro-inflammatory cytokines IL-1β and IL-23 were the most potent cytokines inducing IL-17A production by CD4^+^ T cells and also promoted IFN-γ production (Supplementary Fig. 1a, b). The amount of IL-17A produced by CD4^+^ T cells in response to IL-1β and IL-23 was fivefold higher than that produced by anti-CD3/CD28-stimulated CD4^+^ T cells. However, these pro-inflammatory cytokines had no priming effects on CD4^+^ T cells to produce the T_H_2 cytokine IL-4 (Supplementary Fig. 1c). To further examine which CD4^+^ T-cell subset mainly produced TCR-independent IL-17A and IFN-γ, we isolated naive (CD4^+^CD25^−^CD62L^high^CD44^low^) and memory-like (CD4^+^CD25^−^CD62L^low^CD44^high^) T cells and cultured these cells with IL-7, IL-1β, and IL-23 without anti-CD3/CD28 stimulation. We eliminated the possibility of contamination of innate-like lymphocytes by sorting both naive and memory-like CD4^+^ T cells using NK and γδ TCR^+^ exclusion gates (Supplementary Fig. 2a). We found that IL-1β synergized with IL-23 to promote IL-17A and IFN-γ production by memory-like, but not naive, CD4^+^ T cells in the absence of TCR engagement (Fig. 1a). The frequency of IL-17A single-positive and IL-17A/IFN-γ double-positive CD4^+^ T cells was significantly increased in response to IL-1β, which was further upregulated by adding IL-23 (Fig. 1b, c). In this bystander activation, IL-6 was dispensable for the activation of memory-like CD4^+^ T cells to produce effector cytokines, which are correlated with innate-like lymphocytes^14,32–34^ (Supplementary Fig. 3). Because IL-1β and IL-23 are known to confer pathogenic characteristics to T_H_17 cells^5,7–9^, we assessed the mRNA levels of pathogenic T_H_17-related cytokines and transcription factors in cytokine-primed naive and memory-like CD4^+^ T cells (Fig. 1d). IL-1β promoted T_H_17-related genes, including IL-17A, IFN-γ, IL-22, GM-CSF, and RORγt, in memory-like CD4^+^ T cells (Fig. 1d). IL-1β acted synergistically with IL-23 to prime the bystander-activated memory-like CD4^+^ T cells to produce IL-17A, IFN-γ, and IL-22 (Fig. 1d). Taken together, these findings suggest that memory-like CD4^+^ T cells, but not naive CD4^+^ T cells, secrete IL-17A and IFN-γ in response to the pro-inflammatory cytokines IL-1β and IL-23 in a TCR-independent manner.

### IL-1R1 licenses TCR-independent IL-17A and IFN-γ production

**Fig. 2:**
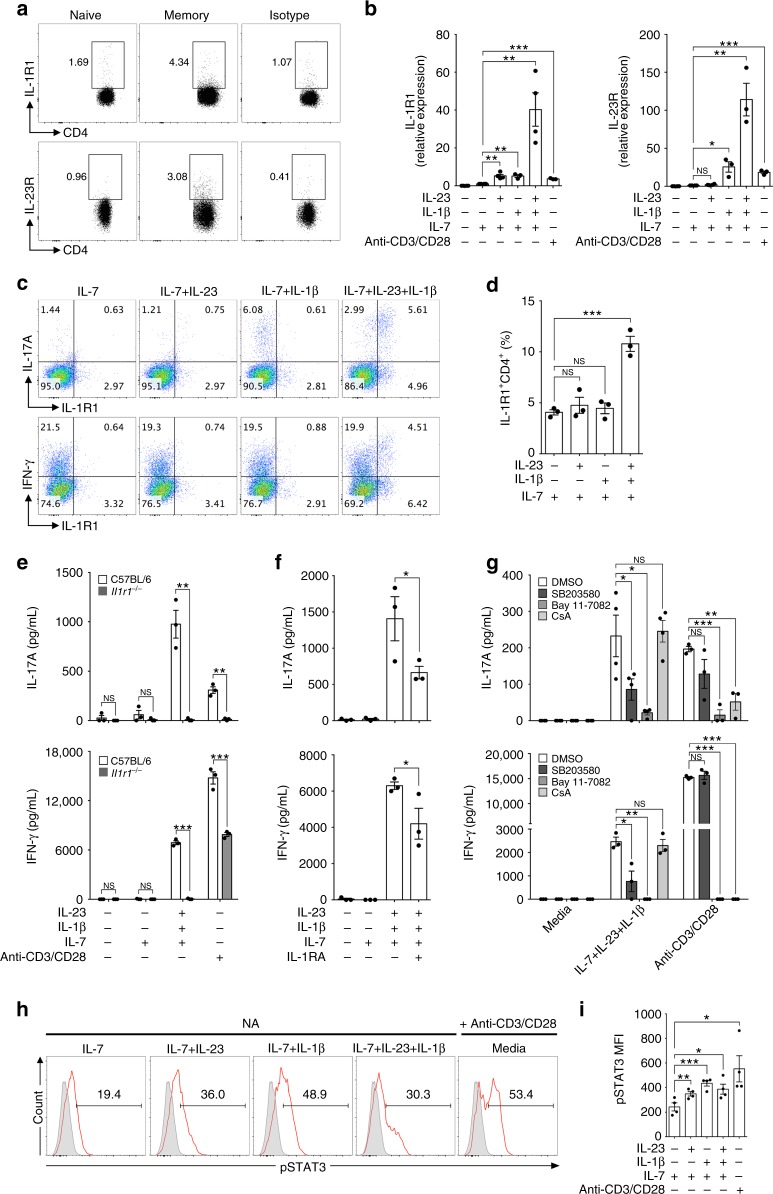
Bystander activation of memory-like CD4^+^ T cells is dependent on IL-1 receptor signaling. **a** The basal expression level of IL-1R1 and IL-23R in naive (CD4^+^CD62L^high^CD44^low^) and memory-like (CD4^+^CD62L^low^CD44^high^) T cells was analyzed by flow cytometry. **b** Memory-like CD4^+^ T cells were stimulated with IL-1β and/or IL-23 in the presence of IL-7 and the mRNA levels of IL-1R1 and IL-23R were analyzed by quantitative real-time PCR. **c**, **d** Memory-like CD4^+^ T cells were stimulated with IL-1β and/or IL-23 in the presence of IL-7 for 5 days and the frequencies of IL-1R1, IL-17A, and IFN-γ were analyzed by flow cytometry. **e** Memory-like CD4^+^ T cells from C57BL/6 wild-type and IL-1R1-deficient (*Il1r1*^-/-^) mice were stimulated with IL-1β and IL-23 in the presence of IL-7 or anti-CD3/CD28 for 5 days, and IL-17A and IFN-γ concentrations in the supernatants were measured by ELISA. Memory-like CD4^+^ T cells were stimulated with IL-1β and IL-23 in the presence or absence of **f** IL-1RA (IL-1R1 antagonist) or **g** SB203580 (p38 inhibitor), BAY 11-7082 (NF-κb inhibitor), and CsA (Cyclosporin A) for 5 days. **h**, **i** phosphorylated STAT3 percentage and mean fluorescence intensity were analyzed by flow cytometry. Data are presented as the mean ± SEM of at least three independent experiments. NS, not significant; *p < 0.05, **p < 0.01, ***p < 0.001

Based on our findings that IL-1β and IL-23 selectively affect memory-like, but not naive CD4^+^ T cells, we first evaluated the expression levels of the receptors for these cytokines. Both IL-1R1 and IL-23R were found to be expressed on memory-like, but not on naive, CD4^+^ T cells (Fig. 2a). We next examined whether IL-1β and IL-23 further upregulate the expression levels of IL-1R1 and IL-23R on memory-like CD4^+^ T cells. IL-1β appeared to be a critical factor for the upregulation of both IL-1R1 and IL-23R, whereas IL-23 alone selectively elevated IL-1R1 mRNA expression (Fig. 2b). However, in the presence of IL-1β, IL-23 significantly increased the expression levels of IL-1R1 and IL-23R, suggesting that IL-1β initiates the TCR-independent bystander activation of memory-like CD4^+^ T cells and that IL-23 propagates this response (Fig. 2b). Moreover, we found that IL-1β, together with IL-23, expanded the number of IL-1R1^+^ memory-like CD4^+^ T cells, which produce IL-17A and IFN-γ (Fig. 2c, d). To confirm the importance of IL-1 in bystander-activated memory-like CD4^+^ T cells, we conducted FACS sorting of memory-like (CD4^+^CD25^−^CD62L^low^CD44^high^) T cells from either *Il1r1*^−/−^ mice or from wild-type C57BL/6 mice, which were stimulated with IL-1β and IL-23. IL-17A and IFN-γ production by memory-like CD4^+^ T cells was significantly decreased in *Il1r1*^−/−^ T cells (Fig. 2e). Furthermore, treatment with the IL-1 receptor antagonist (IL-1RA) reduced IL-17A and IFN-γ production by memory-like CD4^+^ T cells (Fig. 2f), suggesting that cytokine-driven activation of T cells depends on IL-1R1 signaling. Because IL-1β has been reported to trigger the IL-1R1 signaling pathway, which activates signaling proteins, such as mitogen-activated kinases (c-Jun N-terminal kinase (JNK), p38, extracellular signal-regulated kinase (ERK)) and transcription factors (nuclear factor (NF)-κB)^35^, we analyzed the effects of NF-κB inhibitor (Bay11-7082) and p38 inhibitor (SB203580) treatment. The NF-κB inhibitor and p38 inhibitor inhibited IL-17A and IFN-γ production by memory-like CD4^+^ T cells in the absence of anti-CD3/CD28 stimulation (Fig. 2g). Cyclosporin A (CsA) is a calcineurin inhibitor that downregulates nuclear factor-activated T-cell (NFAT) signaling, which is activated by TCR engagement^36^. Importantly, CsA treatment strongly inhibited TCR-stimulated cytokine production, but failed to affect the production of IL-17A and IFN-γ by memory-like CD4^+^ T cells, which is activated by IL-1β and IL-23 without TCR stimulation (Fig. 2g). Because STAT3 signaling is critical for IL-17 production and the development of T_H_17 cells^3,37^, we examined whether IL-1β and IL-23 induce STAT3 activation in the absence of TCR engagement. We found that single or co-treatment of IL-1β and IL-23 directly increased the phosphorylation of STAT3 in memory-like CD4^+^ T cells, showing similar activation by anti-CD3/CD28 (Fig. 2h, i, Supplementary Fig. 4). Collectively, these results demonstrate that IL-1R1 signaling is critical for inducing TCR-independent production of IL-17A and IFN-γ by memory-like CD4^+^ T cells.

### IL-1/IL-23-primed T cells exhibit pathogenic T_H_17 signature

**Fig. 3:**
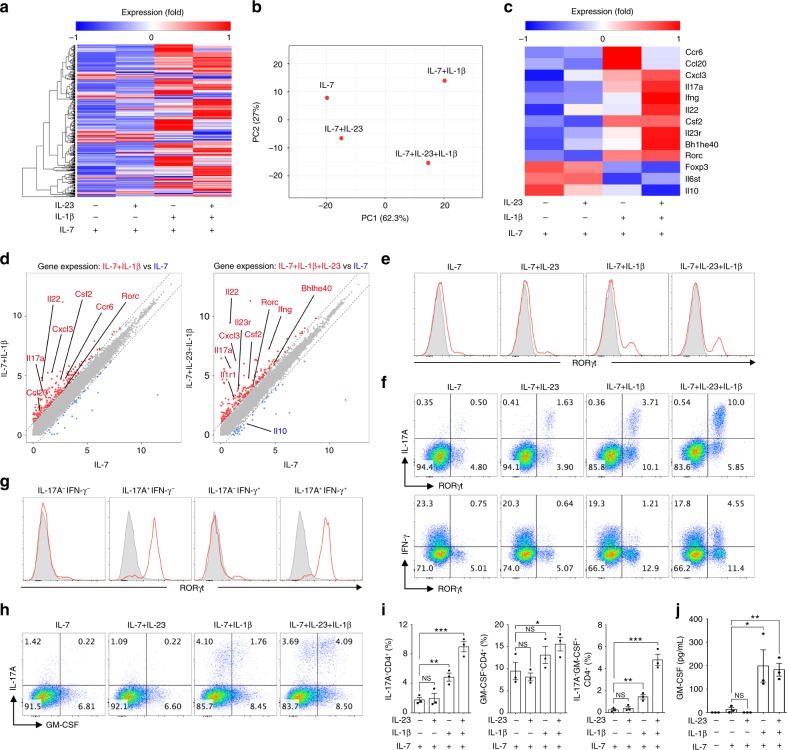
IL-1β and IL-23 induce a pathogenic T_H_17 molecular signature in memory-like CD4^+^ T cells. **a** RNA-seq analysis of 413 genes, upregulated or downregulated by over twofold, in memory-like (CD4^+^CD25^-^CD62L^low^CD44^high^) T cells stimulated in vitro for 5 days with IL-1β and/or IL-23 in the presence of IL-7. **b** Principle component analysis of memory-like CD4^+^ T cells stimulated with IL-1β and/or IL-23 in the presence of IL-7. **c** Heatmap analysis of 13 genes of interest. **d** Scatter plot indicates genes upregulated or downregulated over twofold in memory-like CD4^+^ T cells stimulated with IL-7 versus IL-7 and IL-1β or IL-7 versus IL-7, IL-23 and IL-1β. **e**–**g** FACS-sorted memory-like (CD4^+^CD25^-^CD62L^low^CD44^high^) T cells were stimulated with IL-1β and/or IL-23 in the presence of IL-7 for 5 days. The expression levels of IL-17A, IFN-γ, and RORγt were determined by flow cytometry. **h**, **i** The proportion of IL-17A- and GM-CSF-expressing cells following IL-1 and IL-23 stimulation were analyzed by flow cytometry. **j** GM-CSF concentrations in the culture supernatants were measured by ELISA. Data are presented as the mean ± SEM of three (**e**–**j**) independent experiments. NS, not significant; *p < 0.05, **p < 0.01, ***p < 0.001

To characterize the gene expression profile in bystander-activated memory-like CD4^+^ T cells, we isolated mRNA from memory-like CD4^+^ T cells (CD4^+^CD25^−^CD62L^low^CD44^high^) from C57BL/6 mice and/or stimulated the cells with IL-7, IL-1β, and IL-23. The transcriptomes of the bystander-activated memory-like CD4^+^ T cells were analyzed by RNA sequencing. We identified 413 differentially expressed genes that were upregulated or downregulated by least twofold (Fig. 3a). Of these 413 genes, memory-like CD4^+^ T cells stimulated with IL-1β or with IL-1β and IL-23 in the presence of IL-7 displayed a unique gene expression signature (Fig. 3a, b). Correlating with our reverse transcription (RT)-PCR data, IL-1β upregulated pathogenic T_H_17 signature genes (Cxcl3, Il17a, Ifng, Il22, Csf2, Il23r, Bhlhe40, and Rorc) in a synergistic manner with IL-23 (Fig. 3c). These bystander-activated memory-like CD4^+^ T cells, induced by IL-1β or IL-1β and IL-23, showed decreased expression of non-pathogenic T_H_17 genes, including Foxp3, Il10, and Il6st, which are associated with immune regulation. However, they exhibited increased expression of the C–C chemokine ligand Ccl20 and C–C chemokine receptor Ccr6, both of which are important for the migration and recruitment of pathogenic T_H_17 cells into inflammatory tissues. These patterns are represented as a scatter plot (Fig. 3d). Based on the pathogenic T_Η_17-like gene expression profiles of bystander-activated T cells, we next examined the expression of transcription factor RORγt in these cells. IL-1β directly induced RORγt expression in memory-like CD4^+^ T cells (Fig. 3e). We found that RORγt^high^ memory-like CD4^+^ T cells were the major contributors to IL-17A and IFN-γ production (Fig. 3f). IL-17A single-positive and IL-17A/IFN-γ-double-positive cells expressed high levels of RORγt, indicating that these RORγt^high^ memory-like CD4^+^ T cells are the main bystander-activated subpopulation (Fig. 3g). Additionally, we confirmed that bystander-activated memory-like CD4^+^ T cells produced GM-CSF, which is a critical factor in T_H_17 pathogenicity. Intracellular cytokine staining (Fig. 3h, i) and an enzyme-linked immunosorbent assay (ELISA) (Fig. 3j) demonstrated that stimulation of memory-like CD4^+^ T cells with IL-1β induced GM-CSF secretion, which was further upregulated by adding IL-23. Taken together, these results suggest that bystander-activated memory-like CD4^+^ T cells by IL-1β and IL-23 adopt a pathogenic T_H_17-like profile, which would play a role in T_H_17-type inflammation.

### Bystander T cells contribute to EAE pathogenesis

**Fig. 4:**
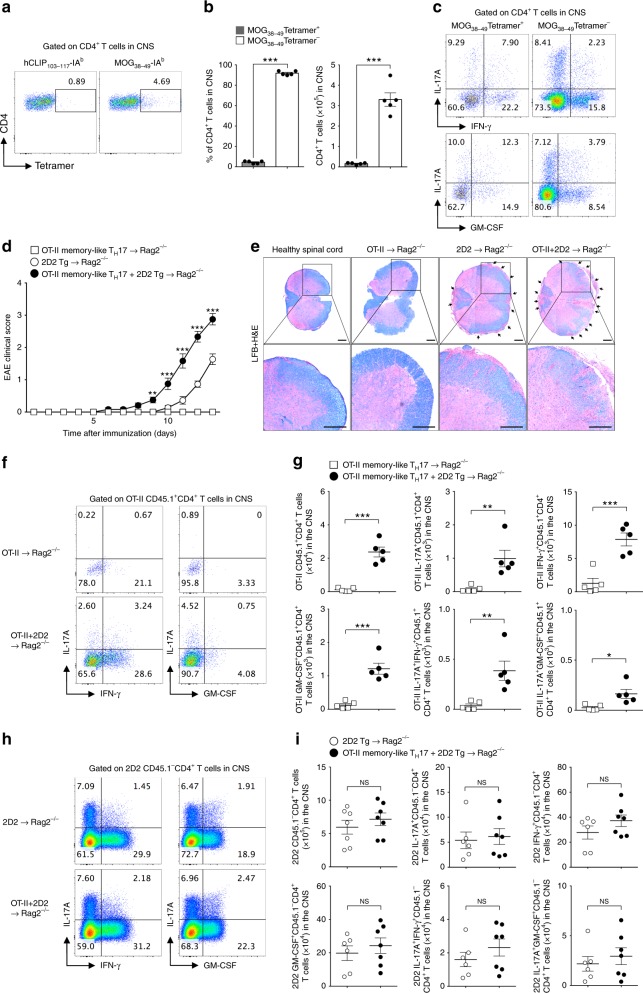
Antigen non-related memory-like T_H_17 cells infiltrate the CNS, amplifying EAE severity. **a**–**c** EAE was induced in 8-week-old female C57BL/6 mice by immunization with MOG in complete Freund’s adjuvant. **a** MOG_38-49-_IA^b^ tetramer staining of CD4^+^ T cells in the spinal cord as determined by flow cytometry. **b** The proportion and absolute cell number of spinal cord-infiltrating CD4^+^ T cells was analyzed at day 14 post-immunization. **c** The proportion of spinal cord-infiltrating cytokine-producing CD4^+^ T cells was analyzed at day 14. **d** Naive CD4^+^ T cells from MOG–TCR-transgenic (2D2) mice were adoptively transferred, with or without in vitro-cultured OT-II memory-like T_H_17 cells, into Rag2^−/−^ mice and immunized with MOG in complete Freund’s adjuvant. Mice EAE clinical scores were monitored daily. **e** Spinal cord tissues were harvested and observed after Luxol fast blue and hematoxylin and eosin staining to determine demyelination and tissue inflammation levels (scale bar, 100 μm). **f** The proportion or **g** absolute cell number of spinal cord-infiltrating cytokine-producing OT-II CD45.1^+^CD4^+^ T cells was analyzed on day 13 after immunization. **h** The proportion or **i** absolute cell number of spinal cord-infiltrating cytokine-producing 2D2 CD45.1^−^ CD4^+^ T cells was analyzed. Data are presented as the mean ± SEM of two (**a**–**c**) or three (**d**–**i**) independent experiments. NS, not significant; *p < 0.05, **p < 0.01, ***p < 0.001

Our previous results raise the possibility that antigen-independent, bystander-activated, memory-like CD4^+^ T cells could play a role in T_H_17-related autoimmune pathogenesis. To further investigate the immunopathological role for this bystander response, we hypothesized that bystander-activated memory-like CD4^+^ T cells contribute to the development of EAE, a T_H_17-mediated autoimmune disease model of multiple sclerosis (MS). MOG_38-49_-IA^b^ tetramer staining showed that in an EAE model, most CNS-infiltrating CD4^+^ T cells were MOG-nonspecific (Fig. 4a, b), which is consistent with the results of previous studies^24–26^. Interestingly, MOG-nonspecific CD4^+^ T cells produced large amounts of IL-17A, IFN-γ, and GM-CSF in the CNS (Fig. 4c). To further examine the role of antigen non-related CD4^+^ T cells in EAE pathogenicity, we generated ovalbumin (OVA)-specific memory-like CD4^+^ T cells in vitro. Naive CD45.1^+^CD4^+^ T cells from ovalbumin–TCR-transgenic (OT-II) mice were primed under T_H_17 conditions for 4 days and further cultured in medium containing IL-7 (Supplementary Fig. 5a). We confirmed the memory phenotypes of these cells on day 14 based on surface marker expression and their lower levels of effector cytokine production (Supplementary Fig. 5b, c). To determine whether OT-II memory-like T_H_17 cells contribute to the development of EAE, we adoptively transferred MOG–TCR-transgenic (2D2) naive CD45.1^-^CD4^+^ T cells, with or without OT-II memory-like T_H_17 cells (CD45.1^+^CD4^+^), into Rag2^−/−^ mice. The mice administered myelin-specific T cells, together with OT-II memory-like T_H_17 cells, developed significantly higher EAE severity (Fig. 4d) and myelin destruction (Fig. 4e) with exaggerated lethality (Supplementary Fig. 6a, b) compared with mice administered myelin-specific naive 2D2 T cells alone. We observed substantial infiltration of antigen non-related OT-II memory-like T_H_17 cells (CD45.1^+^CD4^+^) in the spinal cord, which produced the effector cytokines IL-17A, IFN-γ, and GM-CSF when co-transferred with myelin-specific CD4^+^ T cells (Fig. 4f, g). After demonstrating that antigen non-related OT-II memory-like T_H_17 cells contribute to EAE development, we examined whether these cells affect the infiltration and effector cytokine production of myelin-specific T cells. However, the absolute number of CNS-infiltrating myelin-specific T cells and levels of IL-17A, IFN-γ, and GM-CSF were not significantly different (Fig. 4h, i), suggesting that antigen-specific T-cell function was not affected by antigen non-related memory T cells. As complete Freund’s adjuvant (CFA) challenge alone did not contribute to the peripheral activation of OT-II memory-like T_H_17 cells or myelin-specific 2D2 T cells in the development of EAE (Supplementary Fig. 7), we concluded that bystander memory T-cell function in EAE pathogenesis requires an antigen-specific T-cell response. In summary, these data suggest that antigen non-related bystander-activated memory-like CD4^+^ T cells contribute to EAE pathogenicity by amplifying IL-17A, IFN-γ, and GM-CSF production in the CNS.

### CNS-infiltrating kinetics of bystander T cells in EAE

**Fig. 5:**
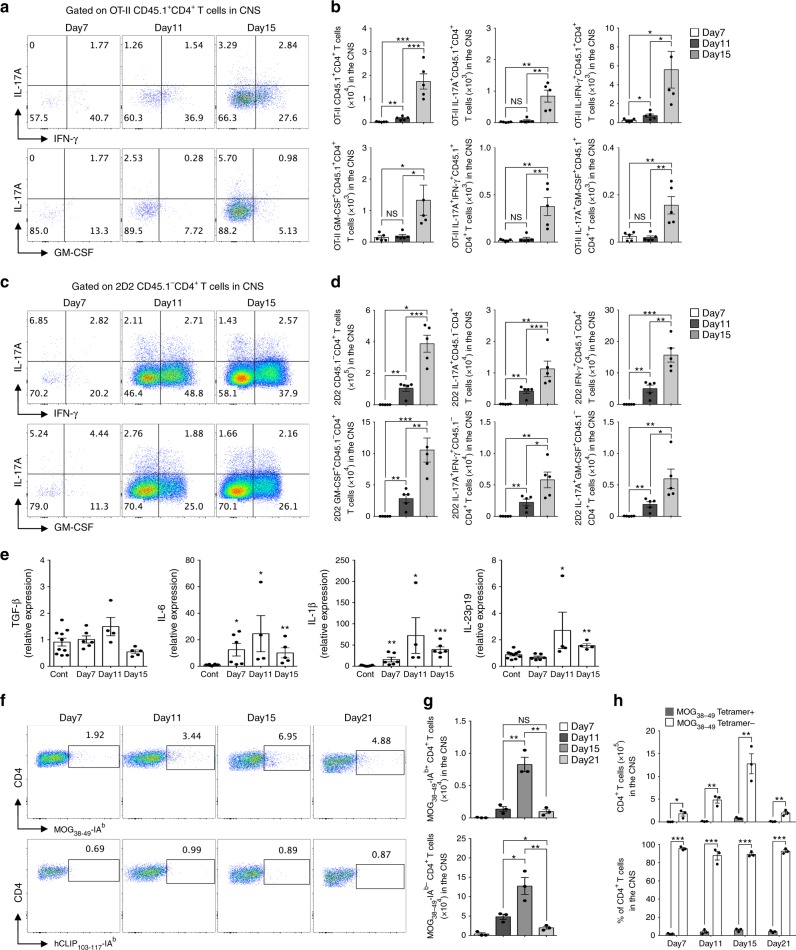
Kinetics of myelin-specific and non-related CD4^+^ T cells infiltration into the spinal cord during the development of EAE. **a**–**d** Naive CD4^+^ T cells from MOG–TCR-transgenic (2D2) mice were adoptively transferred with in vitro-cultured OT-II memory-like T_H_17 cells into Rag2^−/−^ mice and immunized with MOG in complete Freund’s adjuvant. **a** The proportion and **b** absolute cell number of spinal cord-infiltrating cytokine-producing OT-II CD45.1^+^CD4^+^ T cells was analyzed on day 7 (score 0.5–1), day 11 (score 2–2.5), and day 15 (score 3–4) after immunization. **c** The proportion and **d** absolute cell number of spinal cord-infiltrating cytokine-producing 2D2 CD45.1^-^ CD4^+^ T cells was analyzed. **e** The levels of mRNAs encoding cytokines TGF-β, IL-6, IL-1β, and IL-23p19 were analyzed in the spinal cord by quantitative real-time PCR. **f**–**h** EAE was induced in 8-week-old female C57BL/6 mice by immunization with MOG in CFA. **f** MOG_38-49-_IA^b^ tetramer staining of CD4^+^ T cells in the spinal cord as determined by flow cytometry. **g**, **h** The proportion and absolute cell number of spinal cord-infiltrating CD4^+^ T cells was analyzed on day 7 (score 0.5–1), day 11 (score 2–2.5), day 15 (score 3–4), and day 21 (score 1–2) after immunization. Data are presented as the mean ± SEM of two independent experiments. NS, not significant; *p < 0.05, **p < 0.01, ***p < 0.001

Next, we examined the differences in the kinetics of spinal cord infiltration between antigen-specific and non-related T cells during EAE development. We performed identical EAE experiments as described above and checked 2D2 and OT-II T-cell infiltration in the spinal cord and spleen at different time points. We observed significantly increased infiltration of IL-17A^+^, IFN-γ^+^, and GM-CSF^+^-producing OT-II memory-like T_H_17 cells (Fig. 5a, b) along with myelin-specific T cells (Fig. 5c, d) in the spinal cord beginning on day 7, the early onset of EAE, which peaked on day 15. In contrast, the absolute number of 2D2 and OT-II T cells in the spleen gradually decreased by day 15, indicating that both antigen-specific and non-related T cells migrated to the spinal cord from peripheral circulation (Supplementary Fig. 8). We also detected increased mRNA expression of IL-6, IL-1β, and IL-23p19 in the spinal cord tissue during EAE development (Fig. 5e). The IL-1β level was elevated at the onset of clinical symptoms (day 7) and showed greater upregulation during EAE development (days 11 and 15), which coincided with the detection of bystander-activated memory-like T_H_17 cells in the spinal cord. We further confirmed the kinetic display of antigen non-related effector CD4^+^ cells in an active EAE model. MOG_38-49_-IA^b^ tetramer staining revealed that both MOG-specific and MOG-nonspecific CD4^+^ T cells in the spinal cord had comparable kinetic patterns of infiltration (Fig. 5f, g). Although MOG-specific T cells were increased in the spinal cord, MOG-nonspecific T cells infiltrated during EAE, which were dominant over antigen-specific T cells (Fig. 5h). Taken together, these results suggest that antigen non-related CD4^+^ T cells, together with antigen-specific T cells, infiltrate inflammatory regions throughout the development of autoimmune neuroinflammation.

### IL-1R1 is required for bystander T-cell EAE pathogenicity

**Fig. 6:**
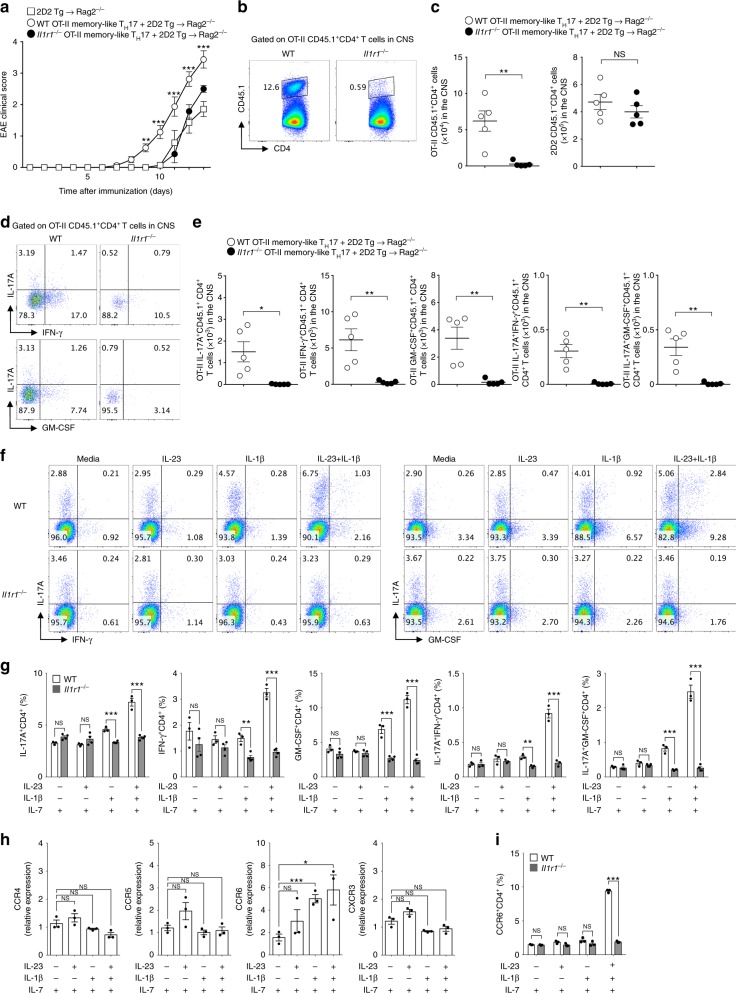
Pathogenic function of antigen non-related memory-like T_H_17 cells is directly mediated by the IL-1 receptor. **a** Naive CD4^+^ T cells from MOG–TCR-transgenic (2D2) mice were adoptively transferred with or without in vitro-cultured OT-II memory-like T_H_17 cells from wild-type or *Il1r1*^−/−^ mice into Rag2^−/−^ mice and were immunized with MOG in complete Freund’s adjuvant. Mice EAE clinical scores were monitored daily. **b** The proportion and **c** absolute cell number of spinal cord-infiltrating CD4^+^ T cells was analyzed on day 13 after immunization. **d** The frequency or **e** absolute cell number of IL-17A, IFN-γ, and GM-CSF was determined by OT-II CD45.1^+^CD4^+^ T cells in the spinal cord. **f**, **g** In vitro-cultured OT-II memory-like T_H_17 cells from wild-type or *Il1r1*^−/−^ mice were stimulated with IL-1β and/or IL-23 in the presence of IL-7 for 3 days. The proportion of IL-17A, IFN-γ, and GM-CSF-producing cells was analyzed by flow cytometry. **h** The levels of mRNAs encoding chemokine receptors were analyzed by quantitative real-time PCR. **i** The proportion of CCR6 expressing cells was analyzed by flow cytometry. Data are presented as the mean ± SEM of two (**a**–**e**) or three (**f**–**i**) independent experiments. NS, not significant; *p < 0.05, **p < 0.01, ***p < 0.001

Based on these results, we next examined whether the pathogenic function of bystander-activated memory-like T_H_17 depends on IL-1R1 signaling. We generated OT-II memory-like T_H_17 cells from wild-type or *Il1r1*^−/−^ mice and confirmed that there were no intrinsic defects in T_H_17 differentiation and memory formation in *Il1r1*^*−/−*^ OT-II cells (Supplementary Fig. 9a, b). OT-II memory-like T_H_17 cells from wild-type or *Il1r1*^−/−^ mice were transferred along with myelin-specific 2D2 T cells into Rag2^−/−^ mice. In the absence of IL-1R1 in OT-II memory-like T_H_17 cells, enhanced susceptibility to the development of EAE was completely lost (Fig. 6a). Although the absolute numbers of CNS-infiltrating myelin-specific T cells were comparable between groups, the number of OT-II memory-like T_H_17 cells in the spinal cord was significantly reduced in the absence of IL-1R1 (Fig. 6b, c). Similarly, a significant reduction was observed in the absolute number and proportion of IL-17A-, IFN-γ-, and GM-CSF-producing OT-II T cells in the spinal cord (Fig. 6d, e). Effector cytokine production by myelin-specific T cells in the spinal cord was not affected by *Il1r1*^−/−^ OT-II memory T_H_17 cells (Supplementary Fig. 10a, b). The transferred *Il1r1*^−/−^ OT-II memory-like T_H_17 cells remained in the spleen compared with the wild-type, with less infiltration into inflammatory tissues (Supplementary Fig. 11). To determine whether effector cytokine production by bystander-activated OT-II memory-like T_H_17 cells depends on IL-1R1 signaling, we cultured in vitro-generated memory-like T_H_17 cells from wild-type or *Il1r1*^−/−^ mice in the presence of IL-1β and IL-23. Importantly, IL-1β directly induced IL-17A, IFN-γ, and GM-CSF production by OT-II memory-like T_H_17 cells, which was enhanced by the addition of IL-23 (Fig. 6f, g). However, *Il1r1*^−/−^ OT-II memory-like T_H_17 cells failed to respond to IL-1β and IL-23, demonstrating the critical role of IL-1R1 signaling in bystander effector function (Fig. 6f, g). Because of the reduced infiltration of *Il1r1*^−/−^ OT-II memory-like T_H_17 cells into the spinal cord, we examined chemokine receptor expression levels. We found that IL-1β directly induced both mRNA (Fig. 6h) and protein expression (Fig. 6i) of the chemokine receptor CCR6, which regulates T_H_17 cell recruitment to the CNS^38^. Thus, the autoimmune pathogenesis of bystander-activated memory-like CD4^+^ T cells appears to depend on IL-1R1 signaling.

### IL-1R1 high human memory T cells produce IL-17A and IFN-γ

**Fig. 7:**
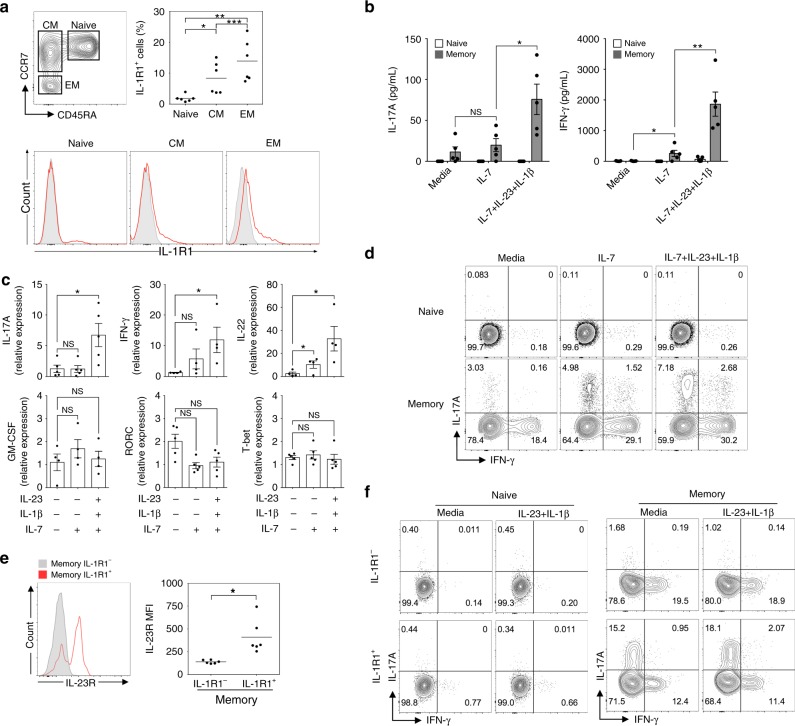
Human IL-1R1^+^ memory CD4^+^ T cells produce IL-17A and IFN-γ in response to IL-1β and IL-23 without TCR stimulation. **a** Flow cytometric analysis of IL-1R1 expression in human naive (CD4^+^CD25^-^CD45RA^+^CCR7^+^), central memory (CM; CD4^+^CD25^-^CD45RA^-^CCR7^+^), and effector memory (EM; CD4^+^CD25^-^CD45RA^-^CCR7^−^) T cells. **b** Human naive and memory (CD4^+^CD25^−^CD45RA^−^CCR7^+/−^) T cells were stimulated with IL-1β and/or IL-23 in the presence of IL-7 for 5 days. IL-17A and IFN-γ concentrations in the supernatants were measured by ELISA. **c** Human memory CD4^+^ T cells were cultured with combinations of IL-1β, IL-23, and IL-7 for 3 days. The levels of mRNAs encoding transcription factors and cytokines were analyzed by quantitative real-time PCR. **d** The frequency of IL-17A and IFN-γ producing human naive and memory CD4^+^ T cells was analyzed by flow cytometry. **e** IL-23R expression in human IL-1R1^+^ and IL-1R1^−^ memory CD4^+^ T cells was analyzed by flow cytometry. **f** IL-1R1^+^ and IL-1R1^−^ cells were sorted from human naive and memory CD4^+^ T cells, stimulated with IL-1β and IL-23 for 5 days, and the frequency of IL-17A and IFN-γ producing cells was determined. Data are presented as the mean ± SEM of six (**a**, **e**) or at least three (**b**–**d**, **f**) independent experiments. NS, not significant; *p < 0.05, **p < 0.01, ***p < 0.001

The observation of antigen non-related bystander activation of memory-like CD4^+^ T cells in mice prompted us to investigate whether human CD4^+^ lymphocytes can produce effector cytokines in a TCR-independent manner. Human central memory (CM; CD4^+^CD25^−^CD45RA^−^CCR7^+^) and effector memory (EM; CD4^+^CD25^-^CD45RA^-^CCR7^−^) T cells showed significantly higher IL-1R1 levels than naive (CD4^+^CD25^−^CD45RA^+^CCR7^+^) T cells (Fig. 7a). Consistent with our results in mice, we found that FACS-sorted human total memory (CD4^+^CD25^−^CD45RA^−^CCR7^+/-^) T cells, but not naive (CD4^+^CD25^−^CD45RA^+^CCR7^+^) T cells, responded to IL-1β and IL-23 to promote IL-17A and IFN-γ production in the absence of TCR engagement (Fig. 7b). The pro-inflammatory cytokines IL-1β and IL-23 increased the mRNA expression of the effector cytokines IL-17A, IFN-γ, and IL-22 in human memory CD4^+^ T cells, while no significant differences were observed in GM-CSF and RORγt levels (Fig. 7c). IL-1β and IL-23 increased IL-17A single-positive and IL-17A/IFN-γ-double positive populations of human memory, but not naive, CD4^+^ T cells (Fig. 7d). Interestingly, IL-1R1^+^ memory CD4^+^ T cells showed higher expression levels of IL-23R (Fig. 7e). Based on these findings, we next FACS-sorted IL-1R1^+^ and IL-1R1^−^ T cells from human memory and naive CD4^+^ T cells and further cultured these cells in medium containing the cytokines IL-1β and IL-23 without anti-CD3/CD28 stimulation. We found that IL-1R1^+^ human memory CD4^+^ T cells were the main subpopulation to be bystander-activated, resulting in the production of IL-17A and IFN-γ (Fig. 7f). Neither IL-1R1^+^ nor IL-1R1^−^ naive CD4^+^ T cells responded to IL-1β and IL-23 (Fig. 7f). Collectively, these data suggest that IL-1R1^+^ human memory CD4^+^ T cells are bystander-activated and produce IL-17A and IFN-γ in the absence of TCR engagement.

## Discussion

We identified a previously unknown innate-like pathogenic function of memory CD4^+^ T cells in autoimmunity. Our findings suggest that antigen non-related memory-like CD4^+^ T cells are an important source of IL-17A and IFN-γ in inflammatory tissues in response to IL-1β and IL-23. Bystander-activated memory-like CD4^+^ T cells express a pathogenic T_H_17 molecular signature, with RORγt, CCR6, and GM-CSF expressed in an IL-1R1-dependent manner. Furthermore, we demonstrated that OVA-specific memory-like T_H_17 cells make an important contribution to EAE pathogenicity regardless of antigen specificity by amplifying IL-17A, IFN-γ, and GM-CSF in the CNS. Our results reveal the importance of antigen non-related memory CD4^+^ T cells in autoimmune disease pathogenesis.

Previous studies showed that antigen non-related CD8^+^ T lymphocytes can be bystander-activated^39–42^. These bystander-activated memory CD8^+^ T cells have been shown to produce IFN-γ in response to IL-12 and IL-18^39^. The ability to produce IFN-γ in the absence of cognate antigen has been demonstrated to contribute to the clearance of infections^39,43^. A recent study showed that antigen non-related bystander-activated CD8^+^ T cells induce an innate-like cytotoxic function, which mediates liver injury in acute hepatitis A^44^. Additionally, in correlation with CD8^+^ T lymphocytes, differentiated T_H_1, T_H_2, and T_H_17 cells have been suggested to have a similar innate-like function in response to IL-1 family cytokines (IL-1, IL-18, and IL-33)^19^. Recently, OVA-specific memory T_H_2 cells were reported to produce IL-13 in an IL-33-dependent manner, and this innate response contributed to allergic inflammation and early helminth infection^20^, suggesting that bystander CD4^+^ T cells have important functions. Previously, we found that IL-18 overexpression in the lung spontaneously induces IFN-γ, IL-13, and IL-17-producing CD4^+^ T cells without exogenous antigen challenge^45^. We next investigated whether CD4^+^ T cells have a critical role in disease pathogenesis in an antigen-independent manner. From our study, we demonstrate an important role for antigen non-related bystander CD4^+^ T cells in EAE pathogenesis, which are activated by IL-1β together with IL-23. These cells are the major source of pathogenic cytokines, including IL-17A, IFN-γ, and GM-CSF, which amplify CNS inflammation in autoimmune disease.

Previously, naive CD4^+^ T cells were shown to express IL-1R1 and IL-23R upon stimulation with TCR and costimulation, and IL-6 further upregulated the expression levels of both the receptors^46,47^. We observed high basal expression level of IL-1R1 and IL-23R in memory-like, but not naive, CD4^+^ T cells, suggesting that antigen-experienced T cells can respond to IL-1β and IL-23 in the absence of TCR stimulation. IL-1 stimulation of memory-like T cells directly upregulated RORγt expression and STAT3 phosphorylation, which induced IL-1R1 and IL-23R expression^46,47^. Importantly, co-treatment with IL-1β and IL-23 significantly increased the expression level of IL-1R1 and IL-23R compared with either cytokine alone, indicating positive feedback between IL-1 and/or IL-23 to affect the expression of their receptors. IL-23 is an essential cytokine for the generation of pathogenic T_H_17 cells^5,7–9^. IL-23 regulates the maintenance of RORγt and induces effector cytokines, including IL-17A, IFN-γ, GM-CSF, and IL-22^5,7–9,48^. Moreover, IL-23 activates the JAK2/STAT3 signaling pathway^49^. These previous reports are consistent with our findings and support that IL-1β is the major cytokine initiating antigen-independent activation of memory-like CD4^+^ T cells and functions synergistically with IL-23 to produce pathogenic T_H_17-related cytokines.

Over the last decade, IL-1 has been shown to play an important role in the pathogenesis of MS. Studies have indicated that IL-1β is highly expressed in MS lesions^50^, and its level in the cerebrospinal fluid is correlated with the MS clinical score^51^. IL-1 was shown to be important for the pathogenic function of encephalitogenic T_H_17 cells. TCR stimulation with IL-23 did not induce IL-17 production by T cells from *Il1r1*^−/−^ mice^52^. In the current study, we found that memory-like CD4^+^ T cells failed to produce effector cytokines in the absence of IL-1R1 signaling, demonstrating the importance of IL-1R1 signaling in both antigen-specific and bystander T-cell activation. T_H_17 cells are classically known to be differentiated by TGF-β1 and IL-6 by producing IL-17A and IL-10, which are not pathogenic^6^. However, further stimulation with IL-1β and IL-23 in T_H_17 cells induces GM-CSF, which is a pathogenic molecule in T_H_17-related diseases^9^. GM-CSF is directly regulated by RORγt and is a critical encephalitogenic factor in the development of EAE^8,9^ and MS^53^. Additionally, IL-1β has been shown to induce Bh1he40, which characterizes pathogenic T_H_17 cells in an EAE model^27^. Through RNA-seq analysis, we also demonstrated that IL-1β increases these pathogenic molecules in memory-like CD4^+^ T cells, which showed a synergistic effect with IL-23, indicating that bystander-activated memory CD4^+^ T cells responding to IL-1, in addition to antigen-specific T cells, is an important pathogenic mechanism of autoimmune neuroinflammation. Interestingly, we confirmed that chemical inhibitors downstream of IL-1R1 signaling inhibited cytokine production by memory-like T cells, which was unaffected by treatment with CsA, a well-known NFAT inhibitory drug^54,55^. CsA may not be sufficient to regulate the pathogenic inflammation, as the inhibition of bystander-activated T cells is independent of calcium signaling.

The interplay between the factors that cause MS is complex. Although previous studies have described various CNS autoantigen-specific T cells in MS patients, none have been identified as the target of MS^56^. Recent studies have highlighted the importance of antigen-nonspecific innate-like lymphocytes such as γδ T cells, invariant natural killer T cells, and innate lymphoid cells in various autoimmune diseases^14–16^. Interestingly, CD4^+^ T cells specific for nonmyelin proteins have been suggested to invade the CNS and have shown encephalitogenic potential^21–24^. One study reported that nonmyelin-specific T cells stimulate the function of antigen-presenting cells and their recruitment into the CNS, which enhances the severity of EAE^22^. These previous studies collectively suggest that antigen non-related CD4^+^ T cells, in conjunction with innate-like lymphocytes, contribute to multiple innate-like pathogenic mechanisms during the development of EAE or MS. Consistent with previous results^24–26^, we observed that most CNS-infiltrating CD4^+^ T cells in the spinal cord of EAE mice were MOG-nonspecific (95.31%). We confirmed that these myelin non-related CD4^+^ T cells contributed to EAE development by amplifying effector cytokines IL-17A, IFN-γ, and GM-CSF in the CNS. Notably, the infiltration of antigen non-related CD4^+^ T cells in the spinal cord showed an identical kinetic pattern as myelin-specific T cells, which synergistically contribute to EAE pathogenicity. We further hypothesized that this cellular infiltration into the CNS depends on the CCR6-CCL20 axis, an important chemokine receptor for the migration of inflammatory T_H_17 cells^38^. We found that IL-1β directly enhances CCR6 expression, suggesting that IL-1 plays a crucial role in antigen non-related memory-like CD4^+^ T cells pathogenicity both by promoting effector cytokine production and by mediating CNS migration. Importantly, a recent study demonstrated that most CNS-infiltrating pathogenic T_H_17 cells expressing Bh1he40 are MOG-nonspecific^27^. Moreover, overexpressing GM-CSF in CD4^+^ T cells spontaneously induces CNS inflammation, regardless of antigen specificity^57^. These reports support our findings that antigen-non-related bystander-activated effector or memory CD4^+^ T cells are actively involved in pathogenic inflammation to amplify or initiate autoimmune disease by producing pathogenic inflammatory mediators, such as IL-17A, IFN-γ, and GM-CSF. We previously demonstrated that the dNP2-ctCTLA-4 protein regulates CNS-infiltrating T_H_1 or T_H_17 cells and inhibits autoimmune encephalomyelitis^58^. We further examined the importance of CTLA-4 signaling in T cells, regardless of antigen specificity. Moreover, in further studies, we will investigate the fine-tune mechanisms of bystander-activated CD4^+^ T cells during the development of autoimmune neuroinflammation, including their interactions with myeloid cells, neuronal cells, etc.

In summary, this is the first study to demonstrate the innate-like pathogenic function of bystander-activated CD4^+^ T cells in autoimmune disease progression driven by IL-1 and IL-23. Our findings highlight the role of antigen non-related T cells in autoimmune diseases, providing a foundation for better understanding the pathogenic mechanisms of autoimmunity and suggesting a new direction for the treatment of autoimmune diseases.

## Methods

### Mice

C57BL/6J, BALB/c, Rag2^−/−^, OT-II TCR-transgenic, 2D2 TCR-transgenic mice were purchased from the Jackson Laboratory (Bar Harbor, ME, USA). IL-1R1-deficient (*Il1r1*^*−/−*^) OT-II CD45.1^+^CD45.2^−^ mice were generated by crossing IL-1R1-deficient (*Il1r1*^*−/*−^) CD45.1^−^CD45.2^+^ with OT-II CD45.1^+^CD45.2^−^ mice. Male and female mice used were between 7 and 12 weeks of age. All mice were maintained in a specific pathogen-free facility at Hanyang University, and all animal protocols used in this study were approved by the Animal Experimentation Ethics Committee of Hanyang University. Experiments were performed according to the guidelines of the Institutional Animal Care and Use Committees of Hanyang University.

### Cell isolation and differentiation

Naive (CD4^+^CD25^−^CD62L^high^CD44^low^) and memory-like (CD4^+^CD25^−^CD62L^low^CD44^high^) T cells from the spleens and lymph nodes of 7–12-week-old mice were isolated using a FACS Aria cell sorter II (BD Biosciences, Franklin Lakes, NJ, USA). Purified naive and memory-like CD4^+^ T cells were stimulated with IL-1β (20 ng/mL, R&D Systems, Minneapolis, MN, USA), IL-23 (20 ng/mL, R&D Systems), IL-7 (10 ng/mL, Peprotech, Rocky Hill, NJ, USA), or plate-bound anti-CD3/CD28 (2 μg/mL, BD Biosciences). Cytokines and other reagents used in further cell culture experiments included TNF (20 ng/mL, Peprotech), IL-12 (10 ng/mL, Peprotech), IL-1RA (100 ng/mL, R&D Systems), Bay 11-7082 (1 μM, Calbiochem, San Diego, CA, USA), SB203580 (1 μM, Calbiochem), and CsA (50 ng/mL, Calbiochem). To generate OT-II memory-like T_H_17 cells in vitro, naive (CD4^+^Vα11^+^CD25^−^CD62L^high^CD44^low^) T cells were sorted from either OT-II or IL-1R1-deficient (Il1r1^−/−^) OT-II mice. These CD4^+^ T cells were primed with anti-CD3/CD28 (4 μg/mL) in the presence of TGF-β (0.5 ng/mL, R&D Systems), IL-6 (30 ng/mL, BD Biosciences), and IL-23 (20 ng/mL, BD Biosciences). After a 4-day priming period, the OT-II T_H_17 cells were washed and further cultured in medium containing IL-7 (10 ng/mL, Peprotech) for 8–10 days.

### Flow cytometry

Cell staining was performed using the following monoclonal antibodies: anti-CD4 (RM4-5; eBioscience, San Diego, CA, USA, dilution 1:500), anti-CD25 (PC61.5; eBioscience, dilution 1:500), anti-CD44 (IM7; BioLegend, San Diego, CA, USA, dilution 1:500), anti-CD62L (MEL-14; BioLegend, dilution 1:500), anti-Vα11 (B20.1; eBioscience, dilution 1:500), anti-Vβ11 (RR3-15; eBioscience, dilution 1:500), anti-CD45 (30-F11, BioLegend, dilution 1:500), anti-CD45.1 (A20; eBioscience, dilution 1:500), anti-CD69 (H1.2F3; eBioscience, dilution 1:500), anti-IL-23R (12B2B64; BioLegend, dilution 1:100), anti-TCRβ (H57-597; eBioscience, dilution 1:500), anti-γδ TCR (eBioGL3; eBioscience, dilution 1:500), anti-NK1.1 (PK136; BioLegend, dilution 1:500), and anti-phospho-STAT3 (pY705; BD Bioscience, 4 μL per sample). For intracellular staining, the cells were first re-stimulated with a cell stimulation cocktail (00-4975-03; eBioscience) for 4 h at 37 °C, and then staining of cell surface markers was performed. After staining of the surface markers, the cells were fixed and permeabilized in Cytofix/Cytoperm (554714; BD Bioscience) for 30 min at 4 °C. Intracellular staining was performed using the following monoclonal antibodies: anti-IL-17A (eBio17B7; eBioscience, dilution 1:200), anti-IFN-γ (XMG1.2; eBioscience, dilution 1:400), anti-GM-CSF (MP1-22E9; BD Biosciences, dilution 1:200), anti-IL-1R1 (35F5; BD Biosciences, dilution 1:100), anti-RORγt (Q31-378; BD Biosciences, dilution 1:100), and IgG1 (R2-34; BD Biosciences). The APC MOG_38–49_-IA^b^ tetramers were provided by the National Institutes of Health Tetramer Core Facility. The CNS cells were incubated with tetramers (dilution 1:100) for 30 min at 37 °C, and then the cell surfaces were stained. Stained cells were detected by flow cytometry (FACS Canto II, BD Bioscience), and data were analyzed using FlowJo software version 10.0.7 (Tree Star, Ashland, OR, USA).

### Experimental autoimmune encephalomyelitis

Naive (CD4^+^Vβ11^+^CD25^-^CD62L^high^CD44^low^) CD45.1^−^ T cells (5.0 × 10^4^) from MOG–TCR-transgenic (2D2) mice were adoptively transferred with or without 5.0 × 10^5^ in vitro-cultured OT-II memory-like T_H_17 cells into Rag2^-/-^ mice. After transfer, the mice were immunized with 100 μg of MOG_35−55_ peptide in complete Freund’s adjuvant (Difco, Detroit, MI, USA). At 0 and 48 h after immunization, the mice were intraperitoneally treated with 200 ng of pertussis toxin (List Biological Laboratories, Inc., Campbell, CA, USA). Animals were scored daily for signs of clinical disease as follows: partially limp tail, 0.5; completely limp tail, 1; limp tail and waddling gait, 1.5; paralysis of one hind limb, 2; paralysis of one hind limb and partial paralysis of the other hind limb, 2.5; paralysis of both hind limbs, 3; ascending paralysis, 3.5; paralysis of trunk, 4; moribund, 4.5; death, 5. On either day 13 or 14, the mice were killed and the lymphocytes in the spinal cord were isolated by Percoll (GE Healthcare, Little Chalfont, UK) density-gradient centrifugation. For histological analysis, paraffin blocks of spinal cord tissues were de-paraffinized and immersed for combination staining (Luxol fast blue and hematoxylin and eosin).

### Human peripheral blood mononuclear cells

This work was approved by the institutional review committee of Yale University. Human peripheral blood was drawn from healthy adult donors after obtaining written informed consent. Human mononuclear cells were prepared from human peripheral blood on FicollPAQUE (GE Healthcare) gradients. Human naive (CD4^+^CD25^−^CD45RA^+^CCR7^+^) and memory (CD4^+^CD25^-^CD45RA^−^CCR7^+/−^) T cells as well as IL-1R1^+^/IL-1R1^−^ naive and memory CD4^+^ cells were purified using a FACS Aria cell sorter II (BD Biosciences) and cultured in the presence of IL-1β (20 ng/mL, R&D Systems), IL-23 (20 ng/mL, R&D Systems), and IL-7 (10 ng/mL, R&D Systems). The antibodies used to detect cell surface and intracellular markers by staining were anti-CD4 (RPA-T4; BioLegend, dilution 1:100), anti-CD25 (M-A251; BioLegend, dilution 1:100), anti-CD45RA (Hl100; BD Bioscience, dilution 1:100), anti-CCR7 (3D12; BD Bioscience, dilution 1:100), anti-IL-1R1 (FAB269P; R&D Systems, 5 µL per sample), anti-IL-23R (FAB14001A; R&D Systems, 5 µL per sample), anti-IL-17A (BL168; BioLegend, dilution 1:100), and anti-IFN-γ (B27; BD Bioscience, dilution 1:200).

### RT-PCR

Total RNA was isolated with an RNeasy Mini kit (Qiagen, Hilden, Germany), and cDNA was synthesized using the ReverTra Ace qPCR RT master mix (Toyobo, Osaka, Japan) according to the manufacturer’s protocol. Quantitative RT-PCR was performed using iQ SYBR Green Supermix (Bio-Rad, Hercules, CA, USA) with a Bio-Rad CFX Connect real-time PCR detection system. The primers used are listed in Supplementary Table 1. The target gene expression levels were normalized to β-actin. The Il-1r1 (Mm00519943_m1) and Il-23r (Mm00434237_m1) primers were from Applied Biosystems (Foster City, CA, USA). The gene expression levels of these two genes were normalized to Gapdh (Mm03302249_g1).

### RNA sequencing

Total RNA was isolated using TRIzol reagent (Invitrogen, Carlsbad, CA, USA). To construct cDNA libraries with the TruSeq RNA library kit (Illumina, Inc., San Diego, CA, USA), 1 µg of total RNA was used. The protocol consisted of polyA-selection of RNA, RNA fragmentation, random hexamer-primed reverse transcription, and 100-nucleotide paired-end sequencing using a HiSeq4000 system (Illumina, Inc.). The libraries were quantified by qPCR according to the qPCR Quantification Protocol Guide, and quality was assessed using a 2100 Bioanalyzer (Agilent Technologies, Santa Clara, CA, USA). Raw reads from the sequencer were pre-processed to remove low-quality sequences as well as adapter sequences before analysis and the processed reads were aligned to the *Mus musculus* genome (mm10) using HISAT (version 2.0.5). The reference genome sequence for *M. musculus* (mm10) and its annotation were downloaded from the UCSC table browser (www.genome.uscs.edu). After alignment, StringTie (version 1.3.3b) was used to assemble the aligned reads into transcripts and estimate their abundance. Sequencing data were expressed as the relative abundance of the gene transcript in each sample, estimated as the fragments per kilobase of exon per million fragments (FPKM) mapped. The FPKM values were normalized with respect to library size, and thus these values can be used to compare the differential gene expression between samples. Statistical analysis was performed to identify differentially expressed genes using the estimates of abundances for each gene in samples. Genes with one more than FPKM value of zero in the samples were excluded. To facilitate log2 transformation, one was added to each FPKM value for the filtered genes. The filtered data were then log2-transformed and subjected to quantile normalization. The statistical significance of the differential expression data was determined using an independent *t* test and fold-change, in which the null hypothesis was that no differences existed among groups. The false discovery rate was controlled by adjusting the *p-*value using a Benjamini–Hochberg algorithm. For the differentially expressed gene set, hierarchical clustering analysis was performed using complete linkage and Euclidean distance as a measure of similarity. All data analysis and visualization of differentially expressed genes was conducted using R (version 3.3.2).

### ELISA

IL-17A, IFN-γ, IL-4, and GM-CSF levels were measured using a Ready-SET-Go ELISA kit (eBioscience) according to the manufacturer’s instructions.

### Statistics

Data were analyzed using a two-tailed Student’s *t* test or a two-way analysis of variance using GraphPad Prism, version 6.0 (GraphPad Software, La Jolla, CA, USA). *P*-values < 0.05 were considered statistically significant.

## Supplementary Information

Supplementary Information

## Data Availability

The total RNA sequencing data have been deposited in the GEO database under the accession code GSE124045. The authors declare that all other data supporting the findings of this study are available within the article and its Supplementary Information files, or from the corresponding author on reasonable request.

## References

[1] Trapp BD, Ransohoff R, Rudick R (1999). Axonal pathology in multiple sclerosis: relationship to neurologic disability. Curr. Opin. Neurol..

[2] Ivanov II (2006). The orphan nuclear receptor RORgammat directs the differentiation program of proinflammatory IL-17+T helper cells. Cell.

[3] Harris TJ (2007). Cutting edge: an in vivo requirement for STAT3 signaling in TH17 development and TH17-dependent autoimmunity. J. Immunol..

[4] Goverman J (2009). Autoimmune T cell responses in the central nervous system. Nat. Rev. Immunol..

[5] Lee Y (2012). Induction and molecular signature of pathogenic TH17 cells. Nat. Immunol..

[6] McGeachy MJ (2007). TGF-beta and IL-6 drive the production of IL-17 and IL-10 by T cells and restrain T(H)-17 cell-mediated pathology. Nat. Immunol..

[7] Ghoreschi K (2010). Generation of pathogenic T(H)17 cells in the absence of TGF-beta signalling. Nature.

[8] Codarri L (2011). RORgammat drives production of the cytokine GM-CSF in helper T cells, which is essential for the effector phase of autoimmune neuroinflammation. Nat. Immunol..

[9] El-Behi M (2011). The encephalitogenicity of T(H)17 cells is dependent on IL-1- and IL-23-induced production of the cytokine GM-CSF. Nat. Immunol..

[10] Brennan PJ, Brigl M, Brenner MB (2013). Invariant natural killer T cells: an innate activation scheme linked to diverse effector functions. Nat. Rev. Immunol..

[11] Vantourout P, Hayday A (2013). Six-of-the-best: unique contributions of gammadelta T cells to immunology. Nat. Rev. Immunol..

[12] Walker JA, Barlow JL, McKenzie AN (2013). Innate lymphoid cells--how did we miss them?. Nat. Rev. Immunol..

[13] Kwon DI, Lee YJ (2017). Lineage differentiation program of invariant natural killer T cells. Immune Netw..

[14] Sutton CE (2009). Interleukin-1 and IL-23 induce innate IL-17 production from gammadelta T cells, amplifying Th17 responses and autoimmunity. Immunity.

[15] Hatfield JK, Brown MA (2015). Group 3 innate lymphoid cells accumulate and exhibit disease-induced activation in the meninges in EAE. Cell. Immunol..

[16] Buonocore S (2010). Innate lymphoid cells drive interleukin-23-dependent innate intestinal pathology. Nature.

[17] Lee JS (2015). Interleukin-23-independent IL-17 production regulates intestinal epithelial permeability. Immunity.

[18] Zhu J, Yamane H, Paul WE (2010). Differentiation of effector CD4 T cell populations (*). Annu. Rev. Immunol..

[19] Guo L (2009). IL-1 family members and STAT activators induce cytokine production by Th2, Th17, and Th1 cells. Proc. Natl Acad. Sci. USA.

[20] Guo L (2015). Innate immunological function of TH2 cells in vivo. Nat. Immunol..

[21] Hickey WF, Hsu BL, Kimura H (1991). T-lymphocyte entry into the central nervous system. J. Neurosci. Res..

[22] Ludowyk PA, Willenborg DO, Parish CR (1992). Selective localisation of neuro-specific T lymphocytes in the central nervous system. J. Neuroimmunol..

[23] Jones RE, Kay T, Keller T, Bourdette D (2003). Nonmyelin-specific T cells accelerate development of central nervous system APC and increase susceptibility to experimental autoimmune encephalomyelitis. J. Immunol..

[24] Lees JR, Sim J, Russell JH (2010). Encephalitogenic T-cells increase numbers of CNS T-cells regardless of antigen specificity by both increasing T-cell entry and preventing egress. J. Neuroimmunol..

[25] Korn T (2007). Myelin-specific regulatory T cells accumulate in the CNS but fail to control autoimmune inflammation. Nat. Med..

[26] Sabatino JJ, Shires J, Altman JD, Ford ML, Evavold BD (2008). Loss of IFN-gamma enables the expansion of autoreactive CD4+T cells to induce experimental autoimmune encephalomyelitis by a nonencephalitogenic myelin variant antigen. J. Immunol..

[27] Lin CC (2016). IL-1-induced Bhlhe40 identifies pathogenic T helper cells in a model of autoimmune neuroinflammation. J. Exp. Med..

[28] Tan JT (2001). IL-7 is critical for homeostatic proliferation and survival of naive T cells. Proc. Natl Acad. Sci. USA.

[29] Kondrack RM (2003). Interleukin 7 regulates the survival and generation of memory CD4 cells. J. Exp. Med..

[30] Chakir H, Lam DK, Lemay AM, Webb JR (2003). “Bystander polarization” of CD4 + T cells: activation with high-dose IL-2 renders naive T cells responsive to IL-12 and/or IL-18 in the absence of TCR ligation. Eur. J. Immunol..

[31] Munk RB (2011). Antigen-independent IFN-gamma production by human naive CD4 T cells activated by IL-12 plus IL-18. PLoS One.

[32] Rachitskaya AV (2008). Cutting edge: NKT cells constitutively express IL-23 receptor and RORgammat and rapidly produce IL-17 upon receptor ligation in an IL-6-independent fashion. J. Immunol..

[33] Doisne JM (2009). Skin and peripheral lymph node invariant NKT cells are mainly retinoic acid receptor-related orphan receptor (gamma)t+and respond preferentially under inflammatory conditions. J. Immunol..

[34] Kim HY (2014). Interleukin-17-producing innate lymphoid cells and the NLRP3 inflammasome facilitate obesity-associated airway hyperreactivity. Nat. Med..

[35] Dinarello CA (2009). Immunological and inflammatory functions of the interleukin-1 family. Annu. Rev. Immunol..

[36] Macian F (2005). NFAT proteins: key regulators of T-cell development and function. Nat. Rev. Immunol..

[37] Mathur AN (2007). Stat3 and Stat4 direct development of IL-17-secreting Th cells. J. Immunol..

[38] Reboldi A (2009). C-C chemokine receptor 6-regulated entry of TH-17 cells into the CNS through the choroid plexus is required for the initiation of EAE. Nat. Immunol..

[39] Berg RE, Crossley E, Murray S, Forman J (2003). Memory CD8+T cells provide innate immune protection against Listeria monocytogenes in the absence of cognate antigen. J. Exp. Med..

[40] Doisne JM (2004). CD8+T cells specific for EBV, cytomegalovirus, and influenza virus are activated during primary HIV infection. J. Immunol..

[41] Kohlmeier JE, Cookenham T, Roberts AD, Miller SC, Woodland DL (2010). Type I interferons regulate cytolytic activity of memory CD8(+) T cells in the lung airways during respiratory virus challenge. Immunity.

[42] Odumade OA (2012). Primary Epstein-Barr virus infection does not erode preexisting CD8(+) T cell memory in humans. J. Exp. Med..

[43] Kastenmuller W, Torabi-Parizi P, Subramanian N, Lammermann T, Germain RN (2012). A spatially-organized multicellular innate immune response in lymph nodes limits systemic pathogen spread. Cell.

[44] Kim J (2018). Innate-like Cytotoxic function of bystander-activated CD8(+) T cells is associated with liver injury in acute hepatitis A. Immunity.

[45] Kang MJ (2012). IL-18 induces emphysema and airway and vascular remodeling via IFN-gamma, IL-17A, and IL-13. Am. J. Respir. Crit. Care. Med..

[46] Chung Y (2009). Critical regulation of early Th17 cell differentiation by interleukin-1 signaling. Immunity.

[47] Yang XO (2007). STAT3 regulates cytokine-mediated generation of inflammatory helper T cells. J. Biol. Chem..

[48] Hirota K (2011). Fate mapping of IL-17-producing T cells in inflammatory responses. Nat. Immunol..

[49] McGeachy MJ (2009). The interleukin 23 receptor is essential for the terminal differentiation of interleukin 17-producing effector T helper cells in vivo. Nat. Immunol..

[50] Cannella B, Raine CS (1995). The adhesion molecule and cytokine profile of multiple sclerosis lesions. Ann. Neurol..

[51] Rossi S (2014). Cerebrospinal fluid detection of interleukin-1beta in phase of remission predicts disease progression in multiple sclerosis. J. Neuroinflamm..

[52] Sutton C, Brereton C, Keogh B, Mills KH, Lavelle EC (2006). A crucial role for interleukin (IL)-1 in the induction of IL-17-producing T cells that mediate autoimmune encephalomyelitis. J. Exp. Med..

[53] Hartmann FJ (2014). Multiple sclerosis-associated IL2RA polymorphism controls GM-CSF production in human TH cells. Nat. Commun..

[54] Martinez-Martinez S, Redondo JM (2004). Inhibitors of the calcineurin/NFAT pathway. Curr. Med. Chem..

[55] Lee JU, Kim LK, Choi JM (2018). Revisiting the concept of targeting NFAT to control t cell immunity and autoimmune diseases. Front. Immunol..

[56] Schmidt S (1999). Candidate autoantigens in multiple sclerosis. Mult. Scler..

[57] Spath S (2017). Dysregulation of the cytokine GM-CSF induces spontaneous phagocyte invasion and immunopathology in the central nervous system. Immunity.

[58] Lim S (2015). dNP2 is a blood-brain barrier-permeable peptide enabling ctCTLA−4 protein delivery to ameliorate experimental autoimmune encephalomyelitis. Nat. Commun..

