# Supplementary material for: Pathogenic function of bystander-activated memory-like CD4+ T cells in autoimmune encephalomyelitis
Source: Nat Commun. 2019 Feb 12;10:709. doi: 10.1038/s41467-019-08482-w (PMC6372661; doi:10.1038/s41467-019-08482-w)
Supplement: Supplementary file 1 — Supplementary Information [file 41467_2019_8482_MOESM1_ESM.pdf]

Pathogenic function of bystander-activated memory-like $CD4^+$ T cells in autoimmune encephalomyelitis

Hong-Gyun Lee et al.

Supplementary Figures

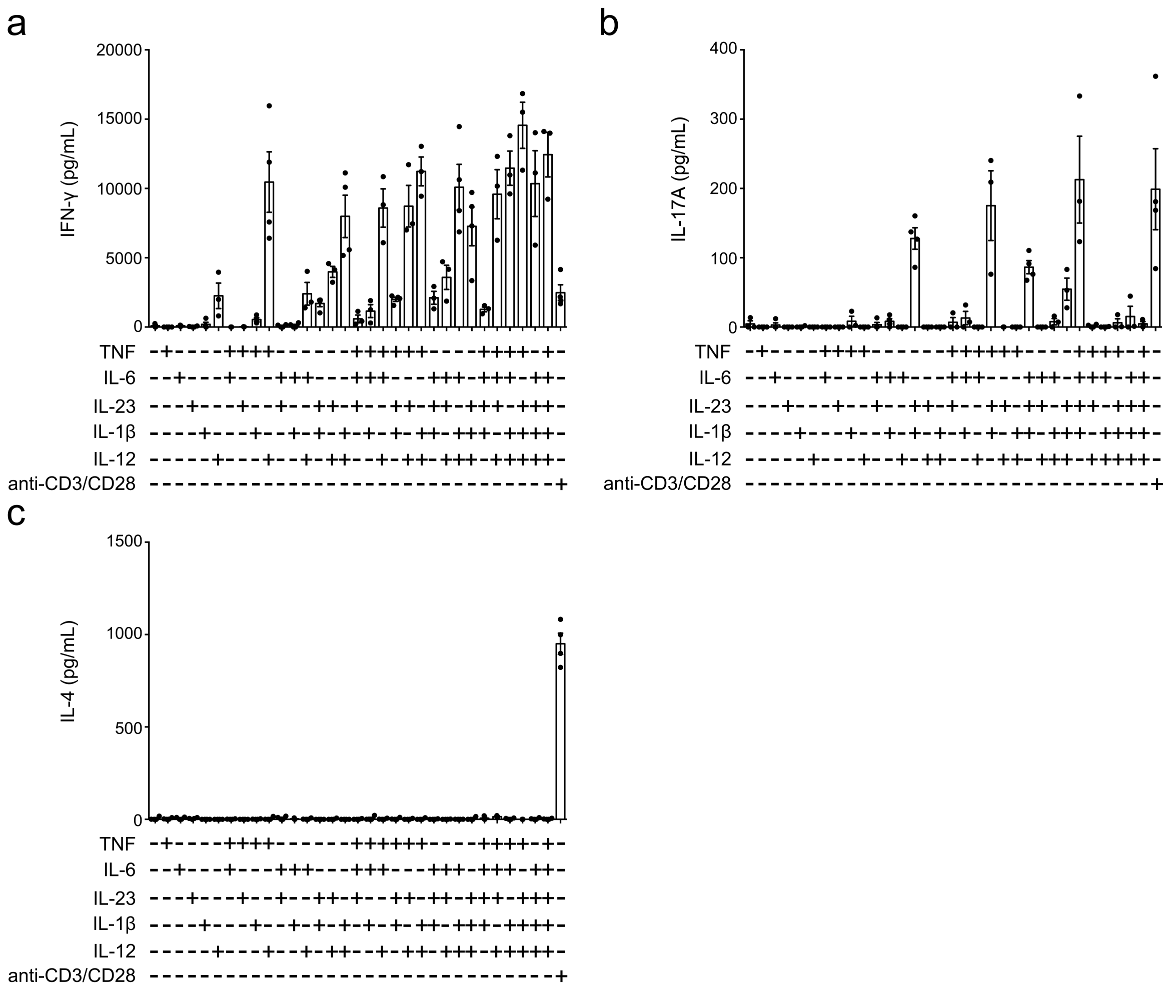

Supplementary Figure 1 Pro-inflammatory cytokines IL-1β and IL-23 promote IL-17A and IFN-γ production by $CD4^{+}CD25^{-}$ T cells in an TCR-independent manner. (**a-c**) FACS-sorted $CD4^{+}CD25^{-}$ T cells were stimulated with a combination of the pro-inflammatory cytokines TNF, IL-6, IL-23, IL-1β, and IL-12 in the presence of IL-7 or anti-CD3/CD28 for 5 days. The production of (**a**) IFN-γ, (**b**) IL-17A, and (**c**) IL-4 was measured by ELISA.

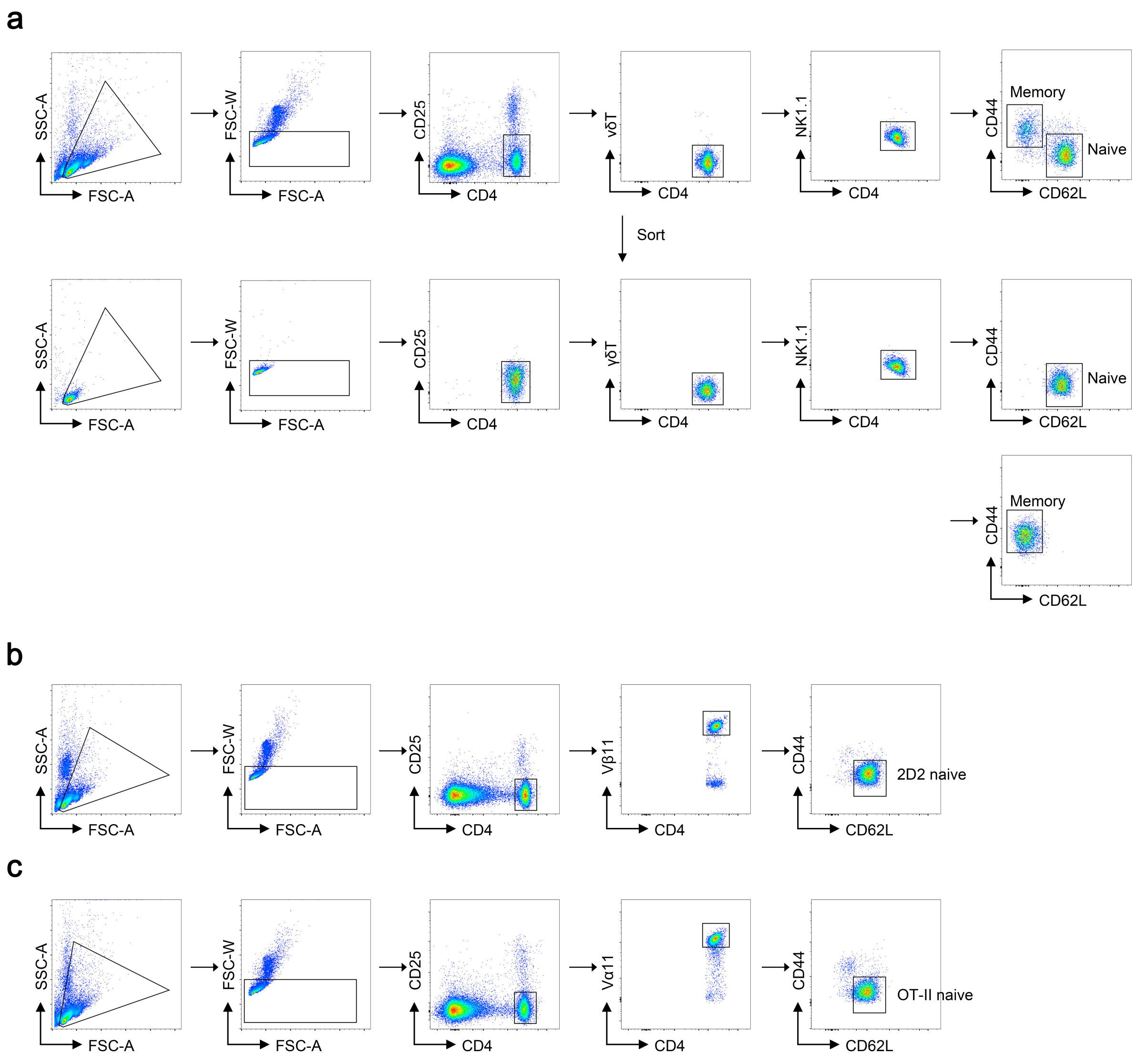

Supplementary Figure 2 FACS gating strategy for cell sorting. (**a**) Spleen and LN cells were stained with fluorescent antibodies against CD4, CD25, γδ TCR, NK1.1, CD44, and CD62L. Naïve ($CD4^{+}CD25$ $CD62L^{high}CD44^{low}$) and memory-like ($CD4^{+}CD25^{-}CD62L^{low}CD44^{high}$) T cells were FACS-sorted for further experiments presented on Figs. 1-3. The black boxes indicate the populations of interest for the outlined studies. (**b**) 2D2 naïve ($CD4^{+}V\beta11^{+}CD25^{-}CD62L^{high}CD44^{low}$) T cells and (**c**) OT-II naïve ($CD4^{+}V\alpha11^{+}CD25^{-}CD62L^{high}CD44^{low}$) T cells were FACS-sorted for further experiments presented on Figs. 4-6.

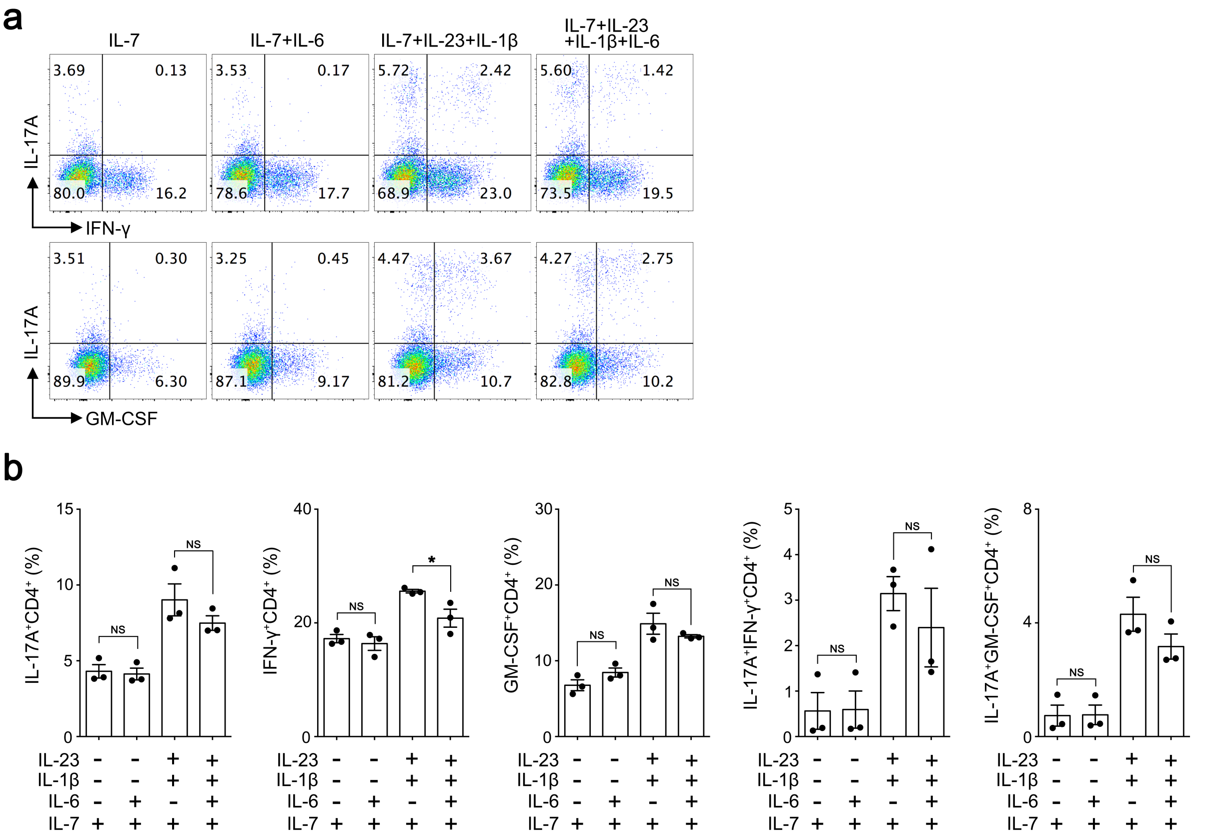

Supplementary Figure 3 IL-6 is dispensable for bystander-activated memory-like CD4$^+$ T cells to promote IL-17A and IFN-γ production. Memory-like (CD4$^+$CD25$^-$CD62L^{low}CD44^{high}) T cells were stimulated with IL-1β, IL-23, and/or IL-6 in the presence of IL-7 for 5 days. (**a, b**) The frequencies of IL-17A and IFN-γ producing cells were analyzed by flow cytometry. Data are presented as mean ± SEM of three independent experiments. NS, not significant; $^*p < 0.05$, $^{**}p < 0.01$, $^{***}p < 0.001$.

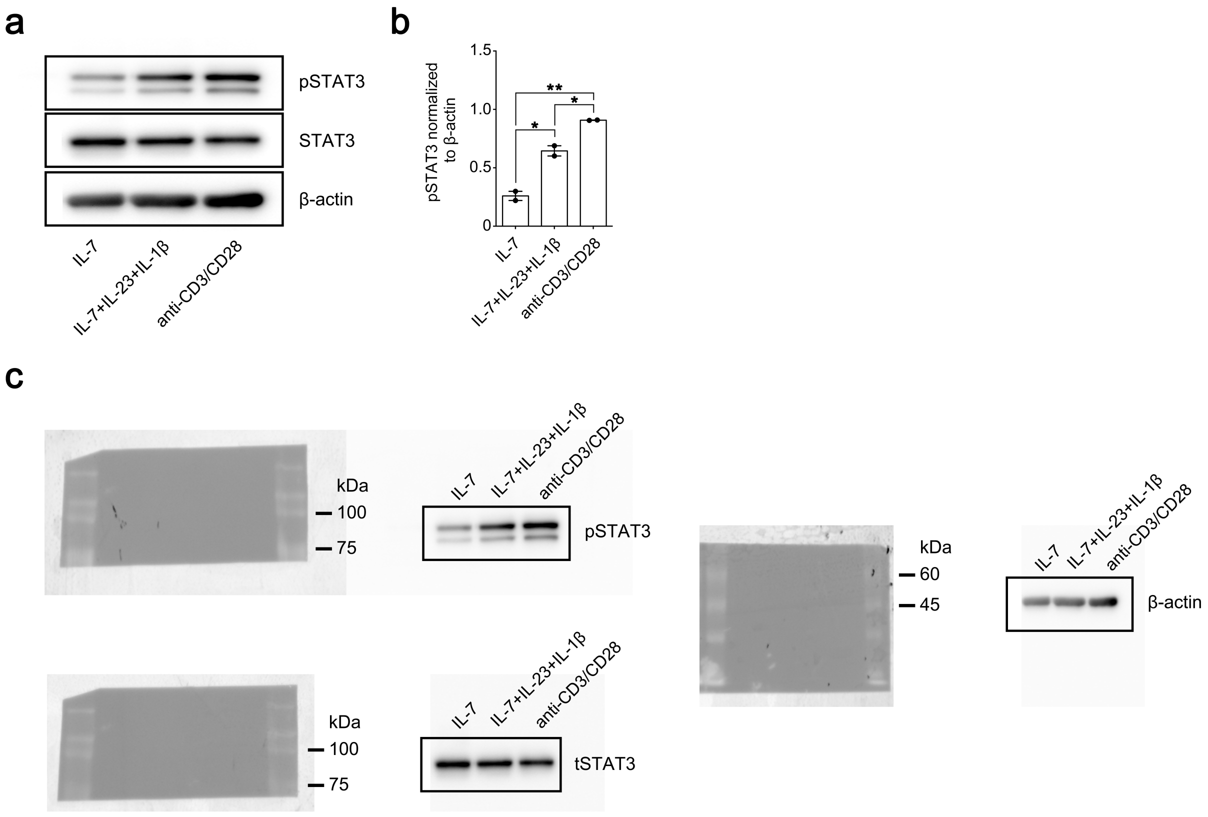

Supplementary Figure 4 IL-1β and IL-23 directly induce STAT3 activation in memory-like CD4$^+$ T cells without TCR engagement. Memory-like (CD4$^+$CD25$^-$CD62L^{low}CD44^{high}) T cells were stimulated with IL-1β and IL-23 in the presence of IL-7 or anti-CD3/CD28 for 5 days. (**a, b**) Phosphorylated STAT3 levels were analyzed by western blot. Densitometric values of band intensity were calculated by normalization to the value of β-actin. Data are presented as mean ± SEM of two independent experiments. NS, not significant; *p < 0.05, **p < 0.01, ***p < 0.001. (**c**) Original immunoblot data corresponding to Supplementary Fig. 4a.

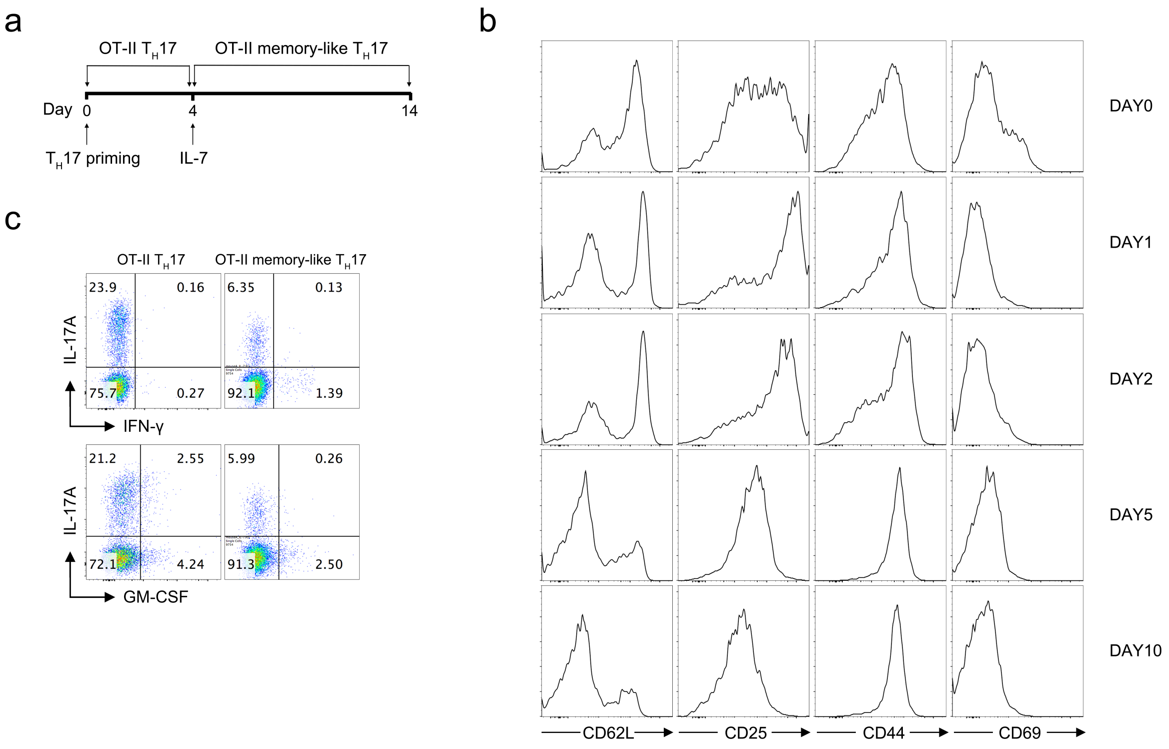

Supplementary Figure 5 Scheme of generating OT-II memory-like T_H17 cells. (**a**) Naïve CD45.1$^+$CD4$^+$ T cells from OVA-TCR-transgenic (OT-II) mice were primed under T_H17 conditions (TGF-β, IL-6, and IL-23) for 4 days, and further cultured in a medium containing IL-7. (**b**) Surface levels of CD62L, CD25, CD44, and CD69 on OT-II memory-like T_H17 cells were analyzed on each day. (**c**) The frequency of IL-17A, IFN-γ, and GM-CSF was determined in OT-II T_H17 (day 4) and OT-II memory-like T_H17 cells (day 14).

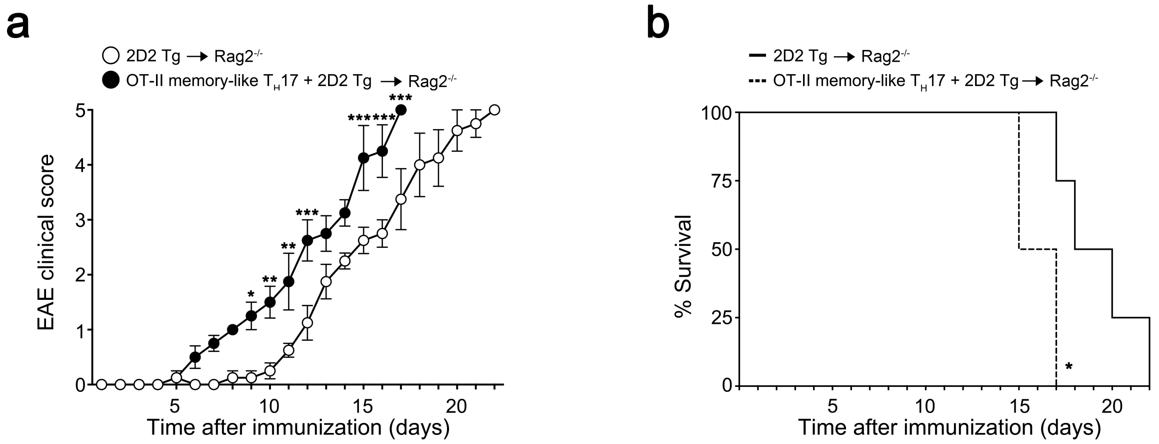

Supplementary Figure 6 Antigen non-related memory-like T_H17 cells contributes to EAE severity and survival. **(a, b)** Naïve $CD4^+$ T cells from MOG-TCR-transgenic (2D2) mice were adoptively transferred with *in vitro*-cultured OT-II memory-like T_H17 cells into $Rag2^{-/-}$ mice and were immunized with MOG in complete Freund's adjuvant (CFA). **(a)** Mice EAE clinical scores were monitored daily. **(b)** Percentage survival of mice during EAE. Data are presented as mean ± SEM of two independent experiments. NS, not significant; *p < 0.05, **p < 0.01, ***p < 0.001.

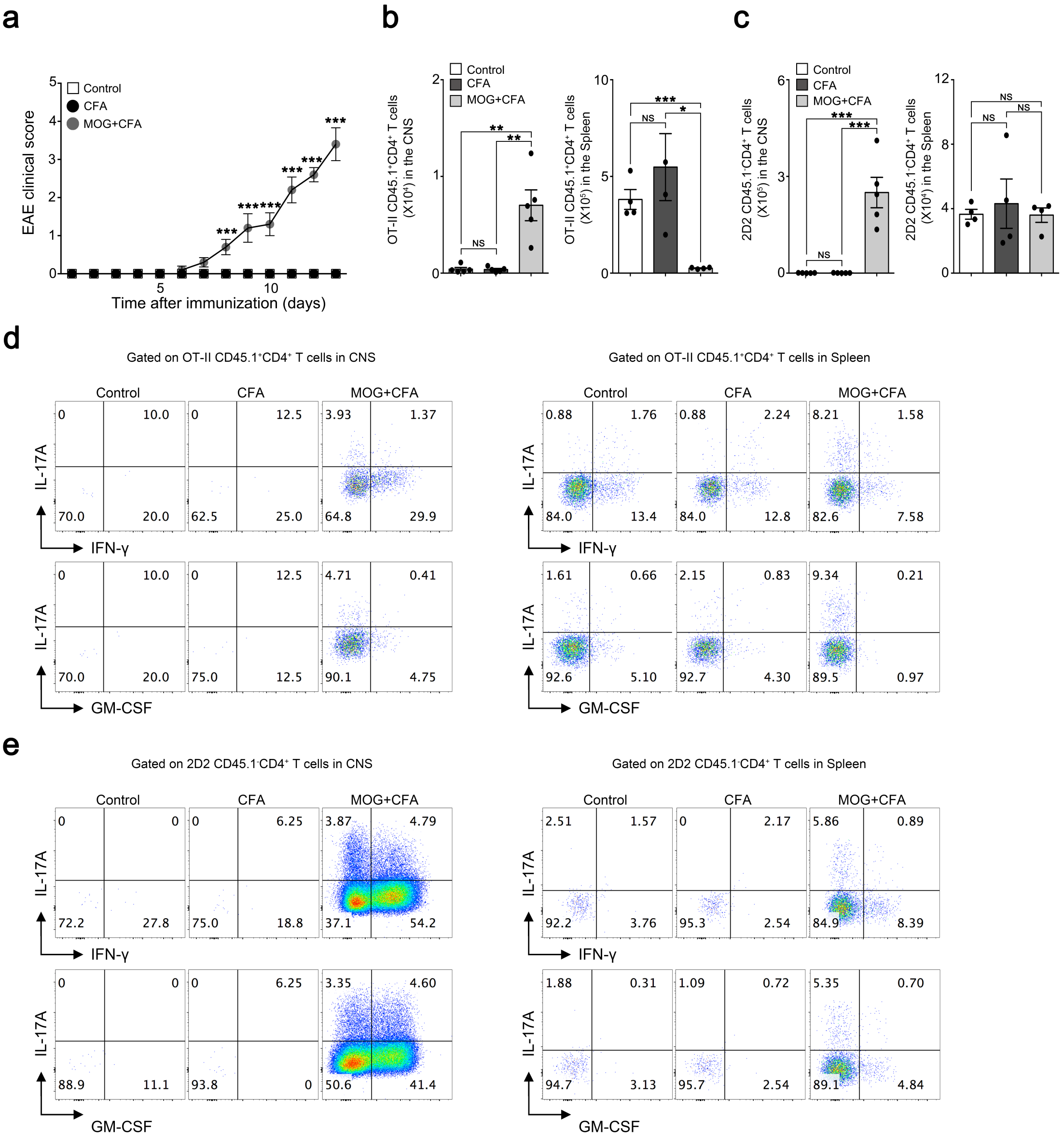

Supplementary Figure 7 Complete Freund's adjuvant (CFA) alone, without antigen immunization, does not induce bystander memory-like T_H17 cells activation during the development of EAE. (**a-e**) Naïve $CD4^+$ T cells from MOG-TCR-transgenic (2D2) mice were adoptively transferred with *in vitro*-cultured OT-II memory-like T_H17 cells into $Rag2^{-/-}$ mice and were immunized with/without MOG and CFA. (**b, c**) The proportion or absolute cell number of OT-II $CD45.1^+CD4^+$ T cells and 2D2 $CD45.1^-$ $CD4^+$ T cells in the spleen and spinal cord was analyzed on day 13 after immunization. (**d, e**) The proportion or absolute cell number of spleen and spinal cord-infiltrating cytokine producing OT-II $CD45.1^+CD4^+$ T cells and 2D2 $CD45.1^-$ $CD4^+$ T cells was analyzed. Data are presented as mean ± SEM of n=5 mice per group. NS, not significant; $*p < 0.05$, $**p < 0.01$, $***p < 0.001$.

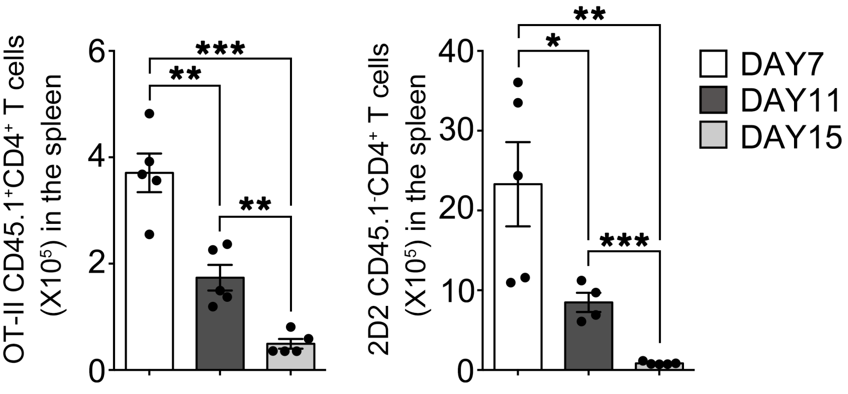

Supplementary Figure 8 Myelin-specific and non-related $CD4^+$ T cells infiltrate to the spinal cord from the peripheral region during the development of EAE. Naïve $CD4^+$ T cells from MOG-TCR-transgenic (2D2) mice were adoptively transferred with *in vitro*-cultured OT-II memory-like T_H17 cells into $Rag2^{-/-}$ mice and were immunized with MOG in complete Freund's adjuvant (CFA). The absolute cell number of spleen-infiltrating OT-II $CD45.1^+CD4^+$ T cells and 2D2 $CD45.1^-$ $CD4^+$ T cells was analyzed on day 7 (score 0.5~1), day 11(score 2~2.5), and day 15 (score 3~4) after immunization. Data are presented as mean ± SEM of two independent experiments. NS, not significant; *$p < 0.05$, **$p < 0.01$, ***$p < 0.001$.

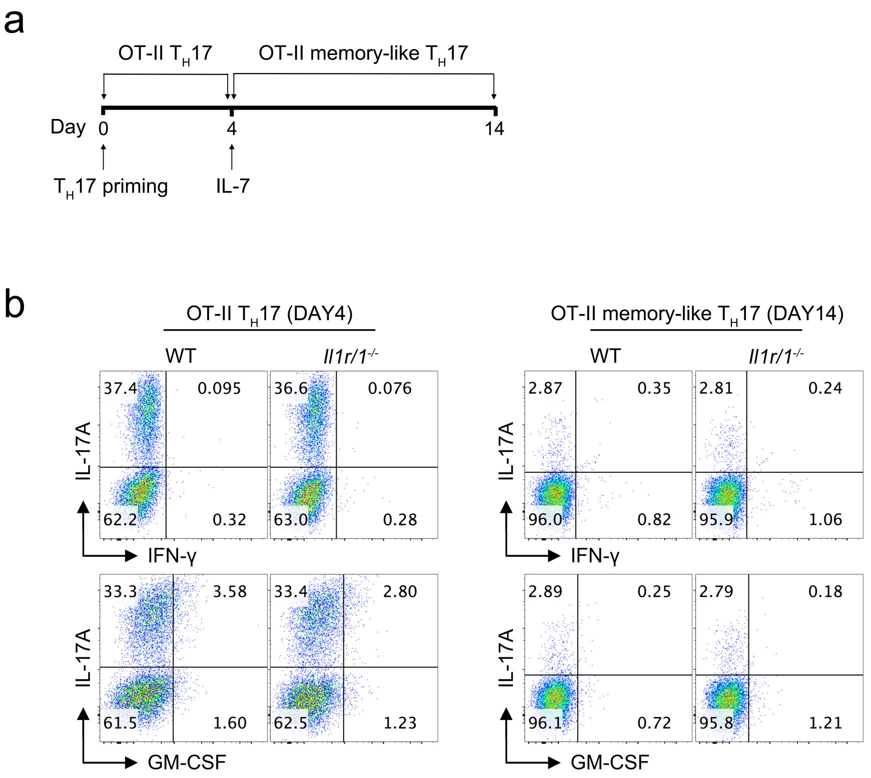

Supplementary Figure 9 *Il1r1*$^{-/-}$ OT-II cells have no intrinsic defect in T_H17 differentiation and memory formation. (**a**) Naïve CD45.1$^+$CD4$^+$ T cells from WT and *Il1r1*$^{-/-}$ OVA-TCR-transgenic (OT-II) mice were primed under T_H17 conditions (TGF-β, IL-6, and IL-23) for 4 days, and further cultured in a medium containing IL-7. (**b**) Frequency of IL-17A, IFN-γ, and GM-CSF was determined in OT-II T_H17 (day 4) and OT-II memory-like T_H17 cells (day 14) from WT and *Il1r1*$^{-/-}$ OVA-TCR-transgenic (OT-II) mice.

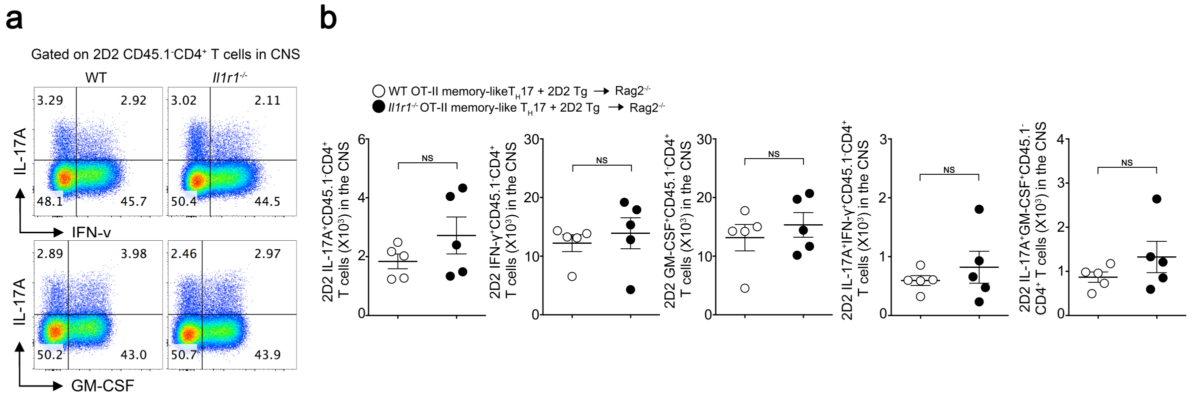

Supplementary Figure 10 *Il1r1*$^{-/-}$ OT-II memory-like T_H17 cells do not affect myelin-specific T cell infiltration and effector cytokine production. Naïve CD4$^+$ T cells from MOG-TCR-transgenic (2D2) mice were adoptively transferred with or without *in vitro*-cultured OT-II memory-like T_H17 cells from wild-type or *Il1r1*$^{-/-}$ mice into Rag2$^{-/-}$ mice and were immunized with MOG in complete Freund's adjuvant (CFA). Spinal cord-infiltrating CD4$^+$ T cells were analyzed on day 13 after immunization. (**a**) The frequency of IL-17A, IFN-γ, and GM-CSF in spinal cord-infiltrating 2D2 CD45.1$^-$CD4$^+$ T cells and (**b**) the absolute number of cytokine-producing cells were analyzed by flow cytometry. Data are presented as mean ± SEM of two independent experiments. NS, not significant; *p < 0.05, **p < 0.01, ***p < 0.001.

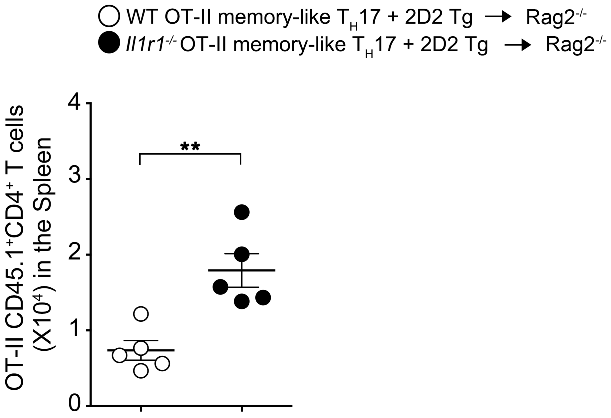

Supplementary Figure 11 *Il1r1*$^{-/-}$ OT-II memory-like T_H17 cells fail to infiltrate into the spinal cord, and remain in the spleen. Naïve $CD4^+$ T cells from MOG-TCR-transgenic (2D2) mice were adoptively transferred, with or without *in vitro*-cultured OT-II memory-like T_H17 cells from wild-type or *Il1r1*$^{-/-}$ mice, into Rag2$^{-/-}$ mice and were immunized with MOG in complete Freund's adjuvant (CFA). The absolute cell number of spleen-infiltrating $CD4^+$ T cells was analyzed on day 13 after immunization. Data are presented as mean ± SEM of two independent experiments. NS, not significant; *p < 0.05, **p < 0.01, ***p < 0.001.

	Forward 5' to 3'	Reverse 3' to 5'
mActb	TGTCCCTGTATGCCTCTGGT	CACGCACGATTTCCCTCTC
mIl17a	TTTAACTCCCTTGGCGCAAAA	CTTTCCCTCCGCATTGACAC
mIfng	ATGAACGCTACACACTGCATC	CCATCCTTTTGCCAGTTCCTC
mIl22	ATACATCGTCAACCGCACCTTT	AGCCGGACATCTGTGTTGTTAT
mCsf2	TGGAAGCATGTAGAGGCCATCA	GCGCCCTTGAGTTTGGTGAAAT
mTbx21	AGCAAGGACGGCGAATGTT	GGGTGGACATATAAGCGGTTC
mRorc	TGCAAGACTCATCGACAAGGC	AGCTTTTCCACATGTTGGCTG
mccr4	GCTCCTCTTACACGCAGTCC	CTTGCCATGGTCTTGGTTTT
mccr5	CGAAAACACATGGTCAAACG	TTCCTACTCCCAAGCTGCAT
mccr6	CCTCACATTCTTAGGACTGGAGC	GGCAATCAGAGCTCTCGGA
mcxcr3	TGCTAGATGCCTCGGACTTT	CGCTGACTCAGTAGCACAGC
mIl6	AGGATACCACTCCCAACAGACCT	CAAGTGCATCATCGTTGTTACTAC
mTgfb1	TCATGTCTCAGTTCCCATCTAGT	GAGAGCGAGGCCATCAGTC
mIl23p19	TGCTGGATTGCAGAGCAGTAA	GCATGCAGAGATTCCGAGAGA
mIl1b	GAAATGCCACCTTTTGACAGTG	TGGATGCTCTCATCAGGACAG
hActb	CGTGGACATCCGCAAAGAC	TGCATCCTGTCGGCAATG
hIl17a	ACCAATCCCAAAAGGTCCTC	GGGGACAGAGTTCATGTGGT
hIfng	CTCTTGGCTGTTACTGCCAGG	CTCCACACTCTTTTGGATGCT
hIl22	GCTTGACAAGTCCAACTTCCA	GCTCACTCATACTGACTCCGTG
hCsf2	CACTGCTGCTGAGATGAATGAAA	GTCTGTAGGCAGGTCGGCTC
hTbx21	GTCCAACAATGTGACCCAGAT	GCTGGTACTTATGGAGGGACTG
hRORC	TTTTCCGAGGATGAGATTGC	CTTTCCACATGCTGGCTACA

Supplementary Table 1. List of primers
